# Using the National Health Interview Survey to understand and address the impact of tobacco in the United States: past perspectives and future considerations

**DOI:** 10.1186/1742-5573-5-8

**Published:** 2008-12-04

**Authors:** Cathy L Backinger, Deirdre Lawrence, Judith Swan, Deborah M Winn, Nancy Breen, Anne Hartman, Rachel Grana, David Tran, Samantha Farrell

**Affiliations:** 1 Division of Cancer Control and Population Sciences, National Cancer Institute, Rockville, MD 20852, USA; 2 Scientific Consulting Group, Inc., Gaithersburg, MD 20878, USA; 3 Tobacco Control Research Branch, Behavioral Research Program, Division of Cancer Control and Population Sciences, National Cancer Institute, 6130 Executive Blvd, MSC 7344, Executive Plaza North, Suite 4038, Rockville, MD 20892-7344, USA

## Abstract

**Objective:**

The National Health Interview Survey (NHIS) is a continuous, nationwide, household interview survey of the civilian noninstitutionalized population of the United States. This annual survey is conducted by the National Center for Health Statistics, part of the Centers for Disease Control and Prevention. Since 1965, the survey and its supplements have provided data on issues related to the use of cigarettes and other tobacco products. This paper describes the survey, provides an overview of peer-reviewed and government-issued research that uses tobacco-related data from the NHIS, and suggests additional areas for exploration and directions for future research.

**Data sources:**

We performed literature searches using the PubMed database, selecting articles from 1966 to 2008. *Study selection*. Inclusion criteria were relevancy to tobacco research and primary use of NHIS data; 117 articles met these criteria. *Data extraction and synthesis*. Tobacco-related data from the NHIS have been used to analyze smoking prevalence and trends; attitudes, knowledge, and beliefs; initiation; cessation and advice to quit; health care practices; health consequences; secondhand smoke exposure; and use of smokeless tobacco. To date, use of these data has had broad application; however, great potential still exists for additional use.

**Conclusion:**

NHIS data provide information that can be useful to both practitioners and researchers. It is important to explore new and creative ways to best use these data and to address the full range of salient tobacco-related topics. Doing so will better inform future tobacco control research and programs.

## Background

Tobacco use is an important health issue in the United States and globally, and its impact on public health has been well documented [1]. Surveillance data on tobacco use and related factors can provide a powerful tool for planning and evaluating population-based prevention and control interventions. One such data system is the National Health Interview Survey (NHIS), a cross-sectional household interview survey conducted by the Centers for Disease Control and Prevention (CDC) through the National Center for Health Statistics (NCHS).

This paper provides the first-ever overview of peer-reviewed and government-issued research conducted using NHIS tobacco-related data. In addition to summarizing and categorizing the findings of these research studies, we describe how the studies have used NHIS data. Summaries of findings are based on published reports by the authors of the papers referenced in this article. We have not attempted to critically review the quality of the analyses or the accuracy of their conclusions. However, by providing this collection of published studies, we hope to encourage researchers to make further use of this valuable data resource in designing studies to help reduce tobacco's toll on public health.

## Analysis

### Data Source: NHIS and Its Supplements

#### Survey overview

NCHS/CDC has administered NHIS annually since 1957 to assess the health of the civilian noninstitutionalized population in the United States. Each year, the NHIS randomly samples about 35,000 households (87,500 persons) from 201 defined geographical units throughout the United States to provide a representative sample of U.S. households. Basic health and demographic information is collected on every member of the household, and in-depth health information is collected on one adult and one child in that household.

#### Survey participation

The households and noninstitutional group quarters selected for interview each week in the NHIS are a probability sample representative of the target population. Survey participation is voluntary and the confidentiality of responses is assured under Section 308(d) of the Public Health Service Act. The NHIS annual response rate is close to 90 percent of the eligible households in the sample.

### Sample design

The description that follows is adapted from Appendices III and VII of the 2006 *NHIS Survey Description *document [2]. Sampling and interviewing for the NHIS are continuous throughout each year. Multistage sampling techniques are used to select the sample of dwelling units for the survey. The sampling plan follows a multistage area probability design that permits the representative sampling of households and noninstitutional group quarters (e.g., college dormitories); the plan is redesigned after every decennial census. In 1997, the survey was substantially redesigned [3], with questions added to capture data on health insurance, access to health care, and health behaviors.

The current sampling plan was implemented in 2006. The first stage of the plan consists of a sample of 428 primary sampling units (PSUs) drawn from about 1,900 geographically defined PSUs that cover the 50 states and the District of Columbia. The multistage methods partition the target universe into several nested levels of strata and clusters. Within a PSU, two types of second-stage units are used: area segments and permit segments. Area segments are defined geographically and contain an expected 8, 12, or 16 addresses. Permit segments cover housing units built after the 2000 census; they are defined using updated lists of building permits issued in the PSU since 2000 and contain an expected four addresses.

The sampling design oversamples Black, Hispanic, and Asian persons. One of the two procedures used for oversampling is "screening." Prior to interviewing, the sample addresses in area segments are randomly separated into two parts. In one part, the sample addresses are assigned to be "screened," and the NHIS interview proceeds through the collection of the household roster. The interview continues only if the household roster contains one or more Black, Hispanic, or Asian persons. Otherwise, the interview terminates and the household is said to be "screened out" In the second part of the NHIS sample, full interviews occur at all households. No screening occurs in permit segments. Another oversampling procedure is applied when area segments are sampled within PSUs. Segments are grouped by 2000 census concentrations of Black, Hispanic, or Asian persons, and groups with higher concentrations are sampled at a higher rate.

### Data collection procedures

Data are collected through a personal household interview conducted by interviewers employed and trained by the U.S. Census Bureau according to procedures specified by NCHS/CDC and detailed in the NHIS report found at:  Nationally, the NHIS uses about 400 interviewers, trained and directed by health survey supervisors in each of the 12 Census Bureau Regional Offices. The revised NHIS questionnaire fielded since 1997 uses a computer-assisted personal interviewing (CAPI) mode. Interviewers use a laptop to administer the CAPI version of the NHIS questionnaire and enter responses as they are given during the interview. This computerized mode of data collection offers distinct advantages in timeliness and improved quality of the data.

### Content of questionnaire

The NHIS questionnaire has two parts: a set of basic health and demographic items – known as the Core Questionnaire – and one or more sets of questions on current health topics. Core Questionnaire questions generally do not vary from year to year, which allows for trend analysis and for pooling data from more than one year to increase sample size for analytic purposes. The Core Questionnaire has four major components: Household, Family, Sample Adult, and Sample Child questionnaires.

The Household questionnaire collects limited demographic information on all individuals living in a house. The Family questionnaire verifies and collects additional demographic information on each family member in the household and data on health status and limitations, injuries, health care access and utilization, health insurance, and income and assets. The Family questionnaire also allows the NHIS to serve as a sampling frame for additional integrated surveys, as needed.

From each family in the NHIS, one sample adult and one sample child (if children are in the household) are randomly selected; information on each is collected with the Sample Adult questionnaire and the Sample Child questionnaire. Because health issues differ for children and adults, some items differ in the two questionnaires. However, both questionnaires collect basic information on health status, health care services, and health behaviors.

Since 1965, the NHIS has included tobacco-related questions – such as questions on cigarette, pipe, and cigar smoking and use of smokeless tobacco (snuff and chewing tobacco) – although methods and questions have varied. Questions on cigarette smoking routinely included in the Core Questionnaire collect data on lifetime smoking status, current smoking status, number of cigarettes smoked per day, age of smoking initiation, and attempts to quit smoking.

In contrast to the Core Questionnaire, NHIS supplements are used to respond to new public health data needs as they arise. These questionnaires may be used to provide additional detail on a subject covered in the Core Questionnaire or on a topic not covered in other parts of the NHIS. Several supplements have included questions on tobacco, such as those designed to assess cancer control and occupational health.

NHIS supplements are administered to a subset of respondents, ranging from 10,000 to 80,000 people. For example, in 1970, questions on smoking were included in a special topic supplement sponsored by the National Cancer Institute (NCI) to explore smoking and health more fully. In one of the more detailed survey supplements, tobacco questions in the 2000 Cancer Control Supplement  focused on the extent of current smoking; smoking cessation; switching to a lower tar and nicotine cigarette; intent to quit smoking; provision of smoking advice from health care professionals; use of other types of tobacco, such as cigars, pipes, chewing tobacco, moist snuff, and bidis; worksite smoke-free policies; home exposure to secondhand smoke; and opinions about smoke-free policies, health effects, and tax increases. These data can be invaluable in assessing the total impact of tobacco use on public health and in identifying strategies to promote healthier lifestyles. Additional file 1 highlights NHIS supplements that have included tobacco questions and provides the survey year, sample size, participant age, and survey topics.

### Strengths and limitations of NHIS data

#### Data strengths

Strengths of the NHIS dataset include its large sample size, large number of variables, and links to other datasets. In addition to being large enough to provide estimates for a number of population subgroups, the data also can be used to compare demographic characteristics – such as gender, age, race/ethnicity, and socioeconomic status – with knowledge, attitudes, and behaviors related to health practices, including tobacco initiation, use, and cessation. The utility of the NHIS dataset is enhanced through links with other NCHS databases that include mortality data, Medicare Enrollment and Claims data, and Social Security Benefit History data. The NHIS also can be linked with Medical Expenditure Panel Survey Linkage Files and the National Immunization Provider Record Check Study (1997–1999).

NHIS questions on tobacco allow researchers to monitor trends in tobacco-related behaviors and can be used to evaluate the context of tobacco use. The criterion of having smoked at least 100 cigarettes (as the threshold for asking additional smoking questions) has been part of the NHIS from the beginning. The wording and positioning of the questions in the interview have been relatively stable. With few exceptions, smoking data are self-reported by the sample adult so that inaccuracies associated with proxy reporting are not an issue. Because tobacco-related questions are embedded in a broad range of questions, NHIS data can be used to relate tobacco behavior to other behaviors and information, such as stress, injury control, cancer screening and knowledge, family history of cancer, alcohol use, dietary knowledge and behaviors, physical activity, health insurance, and social activities. Although such analyses may provide important insights for tobacco prevention and cessation interventions or policies, they have been explored on a limited basis to date. On the other hand, because the NHIS has been used in both research and policymaking arenas for so long, its results can be used as benchmarks.

#### Data limitations

The NHIS does not collect information that may be needed for some tobacco-related research and does not include all subgroup populations. Focusing on health information, the NHIS does not collect data in areas such as labor force participation or industry. Further, the health information collected does not include verifiable medical data or laboratory data, such as blood pressure readings, oximeter readings, or blood and urine data. The NHIS omits institutionalized individuals, thus missing such segments of the population as military personnel or older adults in nursing homes and other long-term care facilities. Also, the age-tobacco use relationships may be biased, as older users may have died before the survey. Finally, because the data from the survey and questions on tobacco are cross-sectional, based on an annual sample, they represent a changing cohort of subjects.

Researchers also need to take into account the limitations inherent in self-reported data, such as that collected by the NHIS. First, it is possible that some respondents may not be forthcoming about a behavior many consider to be undesirable, which could lead to underestimates of current tobacco use and overestimates of attempts to quit such use. The number of cigarettes smoked is subject to the respondents' rounding and estimation error. Information on the age of tobacco initiation depends on the respondents' recall of an event that may not have had a clear starting point and, especially for older respondents, may have occurred a long time ago. Additional file 2 summarizes additional national tobacco-related surveys used in analyzing other variables related to tobacco use, attitudes, knowledge, behaviors, and clinical data.

## Methods

Literature searches through June 2008 in the PubMed database were conducted with the following search terms: "National Health Interview Survey" OR "NHIS" AND "tobacco" or words that began with "smok." The online search began with articles indexed in 1966; articles relating to our search ranged from 1976 to 2008 with 207 journal articles meeting our search criteria. We reviewed articles for relevance to tobacco and excluded those that did not address a tobacco-related question or use the NHIS data directly. Note that our search necessarily resulted in an understatement of all papers that use NHIS data to analyze tobacco use, as not all such papers mention NHIS in their keywords, abstract, or title.

The resulting 117 journal articles are organized in Additional file 3 by the following categories for smoking: prevalence and trends; attitudes, knowledge, and beliefs; initiation; cessation and advice to quit; health care practices; and health consequences. Smokeless tobacco and secondhand smoke are categorized separately. Sociodemographic factors such as gender, age, race/ethnicity, education, occupation, and socioeconomic status cut across all these categories and are addressed, as appropriate, in each area. The categories are not an exhaustive list of all tobacco-related analyses conducted with NHIS data; rather, they represent the main themes of analyses, as determined by the authors.

### Tobacco-related papers using NHIS data

The following summaries of tobacco-related research articles provide an overview of the types of research questions addressed with NHIS tobacco-related data. Discussion of summaries is organized by the categories used in Additional file 3; each discussion concludes with a table that lists the studies within the category of focus. Table details include specific populations, data sources, research questions, report findings and the reference for each study (Additional files 4-11). Note that "specific populations" has been simplified to organize studies for ease of reference; for example, the definition of "adult" or "adolescent" may differ between studies, but the terms reflect the definitions used in the original papers.

We emphasize that the assessment of what each article analyzed and the authors' conclusions, as reported in our tables, is simply a restatement of the original authors' assertions. We did not attempt to critically review the methods or assumptions, check the accuracy of the analysis, or assess the authors' reasoning in drawing their conclusions.

We hope these tables will be used to identify research and/or findings in a field of interest or point to gaps in the research. It is also hoped that the scope of questions and findings reported will suggest additional, valuable avenues of inquiry and potential uses for the analyses conducted. Thus, we encourage readers to review the original papers for details on studies of potential use to them.

#### Prevalence and trends

NHIS data primarily have been used to monitor prevalence and trends of cigarette smoking, as evidenced by the number of publications identified through our literature search. Because the NHIS is conducted annually and the questions vary little from year to year, the survey is one of the key sources of information on trends in cigarette use. These studies also have used NHIS data to explore numerous questions on the prevalence of cigarette smoking, such as how many people smoke cigarettes [4-23], how much they smoke, and tobacco products they use in addition to cigarettes [4,16,24-28]. Questions also have addressed whether smoking prevalence increased or decreased over specified times [15,27,29-31] and examined smoking patterns by sociodemographic factors, such as gender and/or age [32-36], race/ethnicity [37-44], socioeconomic status [9,15,45-49], education [50], and geographic region [23,31,40,51]. In addition, studies have addressed specific populations of interest, such as adolescents [24,35,52-54], adolescents and young adults [55], women [17,45], older adults [10], veterans [56], active military personnel [57], medical professionals [58-60], and other occupational groups [8,45,61]. Many studies have examined combinations of sociodemographic factors and populations of interest. Additional file 4 summarizes the 67 studies that have examined prevalence and trends in the use of cigarettes and other forms of tobacco; the specific populations, data sources, research question, reported findings, and reference are provided for each study.

#### Attitudes, knowledge, and beliefs

Several studies have addressed smoking knowledge, attitudes, and beliefs by examining questions on the beliefs of former smokers [62], knowledge about the health consequences of smoking and about risk factors for smoking or other tobacco use [63-65], and knowledge about oral cancer screening [66,67]. Additional file 5 summarizes the six studies that have examined attitudes, knowledge, and beliefs about tobacco use; the specific populations, data sources, research question, reported findings, and reference are provided for each study.

#### Initiation

Numerous studies have used NHIS data to assess initiation of smoking and factors contributing to smoking initiation, including age [24,53,63,68-70], gender [24,53,63,68,69,71-74], race/ethnicity [9,11,24,25,40,48,49,68,69,71,73-76], and other sociodemographic factors, such as socioeconomic status [49,71] and education [11,26,68,71]. Knowledge of health consequences of smoking [63] also has been analyzed in relation to smoking initiation. Because of the longitudinal nature of the survey, changes in these factors have been studied over time and at particular points in time. Additional file 6 summarizes the 31 studies that have examined the initiation of cigarette smoking; the specific populations, data sources, research question, reported findings, and reference are provided for each study.

#### Cessation and advice to quit

In addition to estimating the prevalence of current and former smokers, NHIS questions allow researchers and policymakers to monitor readiness to quit smoking, cigarette smoking cessation, attempts to quit, economic factors that may influence quit rates, and the extent to which health care providers advise patients to quit. Data from NHIS surveys have been used to look at broad social and economic factors associated with quitting [29,47,77-80] NHIS data also provide insights into whether and how health care professionals – including physicians, family medicine residents, and dentists – advise their patients to quit smoking [10,37,49,53,60,73,78,81-88]. Several studies also have focused on the use of nicotine gum [89], or smokeless tobacco [90,91], as an aid to cessation. Additional file 7 summarizes 23 articles that have examined issues related to tobacco cessation and advice to quit; the specific populations, data sources, research question, reported findings, and reference are provided for each study.

#### Health care practices regarding smoking

A few investigators have used NHIS data to examine the use of health care services and preventive measures by smokers. Several have compared physician and hospital days between smokers and nonsmokers [57,92]. Others look at other health-related behaviors of smokers, such as testing for household radon [93], use of cancer screening exams [94-99], general preventive behaviors [96], and a range of risk behaviors by adolescents [98]. Additional file 8 summarizes the reported findings of these nine studies; the specific populations, data sources, research question, and reference are provided for each study.

#### Health consequences

NHIS data have been used to estimate a range of health consequences. Investigators have reported on the relationship of smoking to other unhealthy behaviors [98], life expectancy [100,101], acculturation [102], premature death [7,103], health care costs [99], lung and heart diseases [42,104], and oral cancer and cancer of the digestive organs [105]. Other studies have taken occupational risks into account when estimating the proportion of lung cancers that are attributable to smoking [106,107]. Additional file 9 summarizes 12 studies that have examined the health consequences of smoking, smokeless tobacco use, and exposure to secondhand smoke; the specific populations, data sources, research question, reported findings, and reference are provided for each study.

#### Secondhand smoke

Numerous studies have analyzed NHIS data in relation to the exposure of nonsmokers, including children, to secondhand smoke (also called environmental tobacco smoke). These studies have assessed exposure to secondhand smoke by children [108], examined health effects on exposed children and adults [109-114], estimated health service use and healthcare expenditures by youth from exposure to secondhand smoke [115], estimated the number of lung cancer deaths attributable to secondhand smoke [103], explored attitudes and beliefs about secondhand smoke [116,117], and considered the confounding effects of toxic chemicals [118]. Additional file 10 summarizes 12 studies that have examined issues related to secondhand smoke; the specific populations, data sources, research question, reported findings, and reference are provided for each study.

#### Smokeless tobacco

The NHIS has provided prevalence and trend data for tobacco products other than cigarettes. Studies have been conducted using data from adults and adolescents and ethnic subpopulations. Gender analysis of smokeless tobacco use also has been conducted. Some of these studies have examined smokeless tobacco in general [4,16,52,86,105,119], while others have compared use of chewing tobacco and snuff [24-27,94] or looked at the use of snuff alone [90,120]. Additional file 11 summarizes the 13 studies that have examined the use of smokeless tobacco products; the specific populations, data sources, research question, reported findings, and reference are provided for each study.

### Applications of the tobacco-related research using NHIS data

#### Improving tobacco surveillance

Scientists have used NHIS data to improve the methodology of tobacco surveillance. The questions used to assess the use of cigarettes and other tobacco products have changed over the years to reflect improvements in the understanding of tobacco use and other tobacco-related variables. For example, in 1992, the definition used to assess self-reported smoking prevalence was modified to specifically include people who smoked only occasionally (some days) because NHIS data revealed a higher prevalence of intermittent smoking than was previously thought to have existed [17]. The category of "current smoking" was therefore changed from "who had smoked 100 cigarettes and smoked now" to "who had smoked 100 cigarettes and now smoke either every day or some days." This new definition is considered to yield a more accurate estimate of current smokers.

#### Supporting analysis of other datasets

Tobacco questions from the NHIS also have been used to assess other methodological issues, including the effect of adjusting for smoking for some occupations [121], estimation of standard errors [120], categorization of educational status [50], reliability of self-reported cigarette consumption [122], and preparation of estimates when combining datasets [123-125]. To most effectively inform research objectives, additional analyses would be of value in assessing methodological differences among surveys, survey designs, and the wording of questions that address similar concepts.

#### Informing programmatic and policy decisions

U.S. government agencies rely on the NHIS to determine the nation's progress toward the Healthy People smoking goals and objectives related to smoking initiation, cigarette use, and cessation patterns. The survey also allows these agencies to monitor success in reaching prevalence or cessation targets. The NHIS provides a national reference population against which other populations can be compared. As such, population-based research allows policymakers and government agencies to assess and predict the impact of social trends or interventions – such as the effect of pricing or smoke-free policies on tobacco use – and to project future burdens of tobacco use.

Results from NHIS studies also provide information on population groups at highest risk for the health consequences of tobacco. Data on subpopulations and underserved populations have been the basis for developing interventions and providing justification for research to better understand tobacco's impact and to develop strategies for addressing the problems these populations face.

#### Supporting other tobacco-related research

Researchers continue to analyze NHIS data to answer a variety of tobacco-related questions for useful information to inform studies. The majority of studies using NHIS data report the prevalence of current and/or former cigarette smoking in the United States and trends of smoking among different U.S. subpopulations. Others look at issues such as the determinants of initiation, use, and cessation; the disease burden of tobacco in the United States; economic and social consequences of tobacco use; and gaps in knowledge about tobacco-related health problems. More recent studies have analyzed data on patterns and trends in the use of quit strategies and on secondhand smoke and tobacco control policies. Analyses of NHIS data also have shed light on how effectively health professionals counsel patients on smoking, the association between cigarette use and other health behaviors, and preventive measures.

In addition to informing future research, program administrators and others in the field can and have used NHIS data to inform important public health activities. However, some analyses that could use NHIS data to inform tobacco programs, policies, and future research have yet to be conducted. For example, the NHIS gathers information on stress, injury control, cancer screening and knowledge, family history of cancer, alcohol use, dietary knowledge and behaviors, physical activity and sun protection behaviors, health insurance, and social activities. These topics have not been explored in the context of tobacco use; such analyses could provide important insight for tobacco prevention and cessation interventions.

## Conclusion

As described in this paper and elsewhere, the NHIS includes national data on the prevalence of tobacco use and a broad range of health issues, such as health conditions, behaviors, utilization of services, health insurance, access to care, injuries, and limitations of activity. Analyses have highlighted trends in cigarette smoking; initiation, use, and cessation rates for other forms of tobacco; secondhand smoke exposure; and correlations between tobacco use and other beliefs and behaviors. Because NHIS data are available for public use , researchers and policymakers are free to explore new and creative ways to use the dataset. Given these trends and the changing climate in which tobacco is being used, these data also can provide insights into tobacco use and its interaction with other health issues. The findings and lessons learned from this type of research have the potential to inform and impact tobacco control research, programs, and policies nationwide.

## Abbreviations

CDC: Centers for Disease Control and Prevention; CAPI: computer-assisted personal interviewing; NCI: National Cancer Institute; NCHS: National Center for Health Statistics; NHIS: National Health Interview Survey; and PSU: primary sampling unit.

## Competing interests

Authors are either employees of the National Cancer Institute (NCI) or are under contract to NCI. The NHIS is funded by the U.S. government and NCI sponsors the cancer and tobacco supplements. NCI presents this paper with the explicit goal of encouraging researchers to develop high-quality research applications that are responsive to the trends and status of tobacco use.

## Authors' contributions

CB provided overall leadership concerning organizational framework of the paper and served as the lead writer of the manuscript. DL provided methodology and background and helped draft the manuscript. JS provided coordination and organization of the literature review and literature summaries. DW participated as the senior epidemiologist in reviewing and drafting the manuscript. NB organized and coordinated the conceptual framework. AH helped draft the manuscript. RG helped draft the manuscript. DT prepared the literature summary tables. SF helped draft the revised manuscript. All authors read and approved the final manuscript.

## Supplementary Material

Additional file 1 **Tobacco-Related Supplements to the National Health Interview Survey (NHIS)**. Click here for file

Additional file 2 **Summary of National Tobacco-Related Surveys**. Click here for file

Additional file 3 **List of Articles That Utilize NHIS Data**. Click here for file

Additional file 4 **Analysis of NHIS Data: Prevalence and Trends**. Click here for file

Additional file 5 **Analyses of NHIS Data: Attitudes, Knowledge, and Beliefs about Tobacco Use**. Click here for file

Additional file 6 **Analyses of NHIS Data: Initiation of Cigarette Smoking**. Click here for file

Additional file 7 **Analyses of NHIS Data: Cessation and Advice to Quit Smoking**. Click here for file

Additional file 8 **Analyses of NHIS Data: Health Care Practices Regarding Smoking**. Click here for file

Additional file 9 **Analyses of NHIS Data: Health Consequences**. Click here for file

Additional file 10 **Analyses of NHIS Data: Secondhand Smoke**. Click here for file

Additional file 11 **Analyses of NHIS Data: Smokeless Tobacco**. Click here for file
