# Supplementary material for: Using the National Health Interview Survey to understand and address the impact of tobacco in the United States: past perspectives and future considerations
Source: Epidemiol Perspect Innov. 2008 Dec 4;5:8. doi: 10.1186/1742-5573-5-8 (PMC2627846; doi:10.1186/1742-5573-5-8)
Supplement: Additional file 1 — Tobacco-Related Supplements to the National Health Interview Survey (NHIS). [file 1742-5573-5-8-S1.doc]

**Table 1. Tobacco-Related Supplements to the National Health Interview Survey (NHIS)**

| **NHIS Supplement**^1,2^ | **Survey Year** | **Sample Size** | **Participant Age** | **Survey Topics** |
| --- | --- | --- | --- | --- |
| **Smoking Supplement** <http://www.cdc.gov/nchs/about/major/nhis/tobacco/nhis_hist_recode_list.htm> | 1970  1978-80 | 35,351  75,497  17,237  10,342 | 17+ | The NHIS Smoking Supplements contain information on smoking status of respondents. Data are also supplied on number of cigarettes smoked, age when started smoking, brands smoked, number of attempts to quit smoking, and tar and nicotine levels of brands smoked. |
| **Cancer Control Supplement (CCS) and Cancer Risk Factor Supplement (CRFS) Epidemiology Study**  **1987:**  [http://wonder.cdc.gov/wonder/sci%5Fdata/surveys/nhis/type%5Ftxt/cnpd87.asp](http://wonder.cdc.gov/wonder/sci_data/surveys/nhis/type_txt/cnpd87.asp)  **1992:**  [http://wonder.cdc.gov/wonder/sci%5Fdata/surveys/nhis/type_txt/canctl92.asp](http://wonder.cdc.gov/wonder/sci_data/surveys/nhis/type_txt/canctl92.asp) | 1987  1992 | 22,043  12,035 | 18+ | The NHIS Cancer Supplements assess cancer prevention behaviors and a variety of demographic and cognitive psychological factors. |
| **Child Health (CH) Supplement**  **1988:** <http://wonder.cdc.gov/wonder/sci_data/surveys/nhis/type_txt/chdhth88.asp>  **1991:**  [http://wonder.cdc.gov/wonder/sci%5Fdata/surveys/nhis/type_txt/child91.asp](http://wonder.cdc.gov/wonder/sci_data/surveys/nhis/type_txt/child91.asp) | 1981  1988  1991 | 17,110 (1988) | 7-17 | Topics covered in the 1988 CH interview include child care, marital history of the child's parents, geographic mobility, circumstances of the pregnancy and birth, injuries, impairments, acute conditions, chronic conditions, passive smoking, sleep habits, school problems, developmental problems, and use of health care services. |
| **Health Promotion/Disease Prevention (HPDP) Supplement** <http://wonder.cdc.gov/wonder/sci_data/surveys/nhis/type_txt/hpdp91.asp> | 1985  1990  1991 | 33,630  41,104  43,732 | 18+ | HPDP assesses prevalence of tobacco use among adults; presence of physician and other health care professional counseling to quit smoking; and smokeless tobacco use. |
| **National Health Interview Survey on Disability (NHIS-D)**  <http://www.cdc.gov/nchs/about/major/nhis_dis/nhis_dis.htm> | 1994  1995 | 107,469 | 5-17 | NHIS-D provides annual estimates of the prevalence of use of selected assistive technology devices for vision, hearing, mobility, and orthopedic impairments, including missing limbs. Also presented are statistics on trends in the prevalence of use of selected mobility assistive technology devices for 1980, 1990, and 1994. |
| **Occupational Health Supplement (OHS)**  [http://wonder.cdc.gov/wonder/sci%5Fdata/surveys/nhis/type_txt/ochlth88.asp](http://wonder.cdc.gov/wonder/sci_data/surveys/nhis/type_txt/ochlth88.asp) | 1988 | 44,233  [6,515 former and nonsmokers analyzed in one example] | 18+ | OHS addresses secondhand smoke in the workplace, discomfort to former and nonsmokers, and workplace restrictions. |
| **Year 2000 Objectives** <http://wonder.cdc.gov/wonder/sci_data/surveys/nhis/type_txt/year2000.asp> | 1993-95 | 21,028  19,738  17,317 | 5+ | Year 2000 objectives include environmental health, tobacco, nutrition, physical activity and fitness, mental health, occupational safety and health, heart disease and stroke, clinical preventive service, family health knowledge, and firearm safety. |
| **Youth Risk Behavior Survey (YRBS)** ^3^  <http://wonder.cdc.gov/wonder/sci_data/surveys/yrbs/type_txt/yrbs92.asp> | 1992 | 10,645 | 12-21 | YRBS collects prevalence data on tobacco use among adolescents. |

^1^ Links for available supplement codebooks can be found under the title of the NHIS supplement. For all supplement codebooks, go to: <http://www.cdc.gov/nchs/about/major/nhis/co-sponsors.htm>.

^2^All supplements are conducted using a household interview method.

^3^ For the YRBS, the household interview is conducted directly with youth after parental permission. Personal audiocassette
