# Supplementary material for: Using the National Health Interview Survey to understand and address the impact of tobacco in the United States: past perspectives and future considerations
Source: Epidemiol Perspect Innov. 2008 Dec 4;5:8. doi: 10.1186/1742-5573-5-8 (PMC2627846; doi:10.1186/1742-5573-5-8)
Supplement: Additional file 2 — Summary of National Tobacco-Related Surveys. [file 1742-5573-5-8-S2.doc]

Table 2. Summary of National Tobacco-Related Surveys

| **Survey** | **Survey Year** | **Sample Size** | **Participant Age** | **Survey Method** | **Survey Topics** |
| --- | --- | --- | --- | --- | --- |
| **National Health Interview Survey (NHIS)** <http://www.cdc.gov/nchs/nhis.htm> | 1957 to present | Varies by year and supplement | 18+ | Household interview | NHIS monitors trends in illness and disability and tracks progress toward achieving national health objectives. |
| **Tobacco Use Supplement of the Current Population Survey (TUS-CPS)**  <http://riskfactor.cancer.gov/studies/tus-cps/> | 1992-93, 1995-96, 1998-99, 2000-03 | 240,000 | 15+ | Telephone/household Interview | TUS-CPS collects national and state-level data on smoking and other tobacco use in the U.S. household population. Questions focus on cigarette and other tobacco prevalence and behavior, medical and dental advice to quit, workplace and home smoking rules/policies, and attitudes and opinions about smoking. |
| **Adult Use of Tobacco Survey (AUTS)**  <http://www.ncbi.nlm.nih.gov/books/bv.fcgi?rid=hstat5.table.11966> | 1964  1966  1970  1975  1986 | 13,031 in 1986 | >21 years in 1964-1975 >17 years in 1986 | Telephone interview | AUTS provides descriptive information on knowledge, attitudes, and behaviors related to tobacco use prevention and control. |
| **Behavioral Risk Factor Surveillance System (BRFSS)**  <http://www.cdc.gov/brfss/about.htm> | 1984-92,  1994-97 | >150,000 | 18+ | Telephone interview | BRFSS collects data on health status, health insurance, routine checkup, diabetes, smoking, pregnancy, women’s health, HIV/AIDS, and demographics. |
| **Hispanic Health and Nutrition Examination Survey (HHANES)**  <http://wonder.cdc.gov/wonder/sci_data/surveys/hanes/hsphanes/type_txt/hspadult.asp> | 1982-85 | 9,643 | 12-74 | **Adolescent and adult history questionnaire** | HHANES provides estimates of the health of Hispanics in general and specific data for Puerto Ricans, Mexican-Americans, and Cuban-Americans. |
| **Health Promotion Survey (HPS), by Statistics Canada for Department of Health and Welfare, currently Health Canada**  <http://www.statcan.ca/cgi-bin/imdb/p2SV.pl?Function=getSurvey&SDDS=3828&lang=en&db=IMDB&dbg=f&adm=8&dis=2> | 1985 | 11,181 | Adults | **Household interview** | HPS surveys current attitudes and behaviors on health and related topics, including physical condition, smoking habits, alcohol and drug consumption, exercise, etc. |
| **National Health and Nutrition Examination Survey (NHANES) Epidemiological Followup Survey (NHEFS)** <http://www.cdc.gov/nchs/about/major/nhefs/nhefs.htm> | 1982-84 | 3,980 | 25-74 | Tracing the cohort of all persons who completed a medical examination at NHANES I in 1971-75 | NHEFS conducts personal interviews with subjects or their proxies; measures pulse rate, weight, and blood pressure of surviving participants; collects hospital and nursing home records of overnight stays; and collects death certificates of decedents. |

| **Survey** | **Survey Year** | **Sample Size** | **Participant Age** | **Survey Method** | **Survey Topics** |
| --- | --- | --- | --- | --- | --- |
| **National Medical Expenditure Survey (NMES), by the Agency for Health Care Policy and Research**  <http://www.icpsr.umich.edu/cocoon/ICPSR/SERIES/00045.xml> | 1987 | About 35,000 individuals in 14,000 households | All | Household interview | NMES collects information on health expenditures by or on behalf of families and individuals, the financing of these expenditures, and each person's use of services. |
| **National Mortality Followback Survey (NMFS)**  <http://www.cdc.gov/nchs/about/major/nmfs/nmfs.htm> | 1986 | 18,733 | 25+ | Death certificates; proxy respondent survey questionnaire; staff person (nursing home and hospice) questionnaire; facility abstract record | NMFS collects socioeconomic status and mortality; associations between risk factors and mortality; and health care sought and provided in the last year of life. |
| **Ontario Health Survey (OHS), by the Ontario Ministry of Health and Long-Term Care, Canada** | 1990 | 49,164 | 12+ | Household interview | OHS is focused on broad aspects of health, including lifestyle behaviors, health problems, and health care utilization. |
| **Monitoring the Future (MTF) Survey**  <http://www.monitoringthefuture.org/> | 1976-2000 | 15,419-18/667/grade in 1998 | Grade12, Grades 8-10 since 1991 | Self-administered, school-based survey | MTF is a study of the behaviors, attitudes, and values of American secondary school students, college students, and young adults. |
| **National Survey on Drug Use & Health (NSDUH), formerly called the**  **National Household Survey on Drug Abuse (NHSDA)**  <http://www.oas.samhsa.gov/nhsda.htm> | 1974, 1976-77, 1979, 1982, 1985, 1988, 1990-98 | 25,500 in 1998 | >12 | In-person household interview; beginning in 1994-B, self-administered answer sheet for responses to sensitive questions | NSDUH provides data on prevalence and correlates of substance use, serious mental illness, related problems, and treatment in the civilian population. |
| **National Youth Tobacco Survey (NYTS)**  <http://www.cdcfoundation.org/annualreport/2000/survey.aspx> | 1999, 2002 | 12,061 in 1999 | Grades 6-12 | Self-administered, school-based survey | NYTS provides data on the factors that contribute to changes in prevalence of tobacco use, such as attitudes and knowledge, exposure to tobacco and secondhand smoke, desire for cessation programs, and a school curriculum with anti-smoking messages. |
| **Teenage Attitudes and Practices Surveys (TAPS I, TAPS II)**  <http://wonder.cdc.gov/wonder/sci_data/surveys/nhis/type_txt/tapsii.asp> | 1989, 1993 | 9,965 in 1989; 4,992 in 1993; 7,960 in longitudinal component | 12-18 in 1989;  10-22 in 1993 | Computer-assisted telephone interview; in-person interview; mailed questionnaire | TAPS is a follow-up study to the NHIS and collects information on attitudes and practices around tobacco in a subsample of NHIS respondents. |
