# Supplementary material for: Using the National Health Interview Survey to understand and address the impact of tobacco in the United States: past perspectives and future considerations
Source: Epidemiol Perspect Innov. 2008 Dec 4;5:8. doi: 10.1186/1742-5573-5-8 (PMC2627846; doi:10.1186/1742-5573-5-8)
Supplement: Additional file 3 — List of Articles That Utilize NHIS Data. [file 1742-5573-5-8-S3.doc]

Table 3: List of Articles That Utilize NHIS Data

| **First Author** | **Article Title** | **Journal** | **Pub Year** | **NHIS Data Year** | **Cigarette Smoking** | | | | | | **Other** | |
| --- | --- | --- | --- | --- | --- | --- | --- | --- | --- | --- | --- | --- |
|  |  |  |  |  | **Prevalence and Trends** | **Attitudes, Knowledge, and Beliefs** | **Initiation** | **Cessation and Advice to Quit** | **Health Care Practices** | **Health Consequences** | **Secondhand Smoke** | **Smokeless Tobacco** |
|  |  |  |  |  |  |  |  |  |  |  |  |  |
| Abraido-Lanza | Do healthy behaviors decline with greater acculturation? Implications for the Latino mortality paradox. | *Soc Sci Med* | 2005 | 1991 |  |  |  |  |  | **X** |  |  |
| Adams | Health-risk behaviors among our Nation's youth: United States, 1992. | *Vital Health Stat 10* | 1995 | 1992 | **X** |  | **X** |  |  |  |  | **X** |
| Anonymous | Cigarette smoking among U.S. adults, 1985-1990, and smoking among selected occupational groups, 1990. | *Stat Bull Metrop Insur* | 1992 | 1985, 1990 | **X** |  |  |  |  |  |  |  |
| Bao | Is some provider advice on smoking cessation better than no advice? An instrumental variable analysis of the 2001 National Health Interview Survey. | *Health Serv Res* | 2006 | 2001 |  |  |  | **X** |  |  |  |  |
| Baranowski | Ethnic differences in cancer risk behaviors through the transition out of high school. | *Ethn Dis* | 1999 | 1992 | **X** |  | **X** |  |  |  |  | **X** |
| Barnes | Health characteristics of the Asian population: United States, 2004-2006 | *Adv Data* | 2008 | 2004-2006 | **X** |  |  |  |  |  |  |  |
| Bonham | Children's health in families with cigarette smokers. | *Am J Public Health* | 1981 | 1970 |  |  |  |  |  |  | **X** |  |
| Boyd | Tobacco use by adults--United States, 1987. | *MMWR Morb Mortal Wkly Rep* | 1989 | 1987 | **X** |  |  |  |  |  |  | **X** |
| Brackbill | Smoking characteristics of US workers, 1978-1980. | *Am J Ind Med* | 1988 | 1978-1980 | **X** |  |  |  |  |  |  |  |
| Breslow | Americans' knowledge of cancer risk and survival. | *Prev Med* | 1997 | 1992 |  | **X** |  |  |  |  |  |  |
| Browning | Socioeconomic disparity in provider-delivered assistance to quit smoking | *Nicotine Tob Res* | 2008 | 2001 |  |  |  | **X** |  |  |  |  |
| Byrd | Predictors of early grade retention among children in the United States. | *Pediatrics* | 1994 | 1988 |  |  |  |  |  |  | **X** |  |
| CDC,NCHS | Health-risk behaviors among persons aged 12-21 years--United States, 1992. | *MMWR Morb Mortal Wkly Rep* | 1994 | 1992 | **X** |  |  |  |  |  |  | **X** |
| CDC,NCHS | Radon testing in households with a residential smoker--United States, 1993-1994. | *MMWR Morb Mortal Wkly Rep* | 1999 | 1993, 1994 |  |  |  |  | **X** |  |  |  |
| CDC,OSH | Cigarette smoking among adults--United States, 1990. | *MMWR Morb Mortal Wkly Rep* | 1992 | 1990 | **X** |  |  |  |  |  |  |  |
| CDC,OSH | Cigarette smoking among adults--United States, 1991. | *MMWR Morb Mortal Wkly Rep* | 1993 | 1991 | **X** |  |  |  |  |  |  |  |
| CDC,OSH | Cigarette smoking among adults--United States, 1992, and changes in the definition of current cigarette smoking. | *MMWR Morb Mortal Wkly Rep* | 1994 | 1992 | **X** |  |  |  |  |  |  |  |
| CDC,OSH | Cigarette smoking among adults--United States, 1993. | *MMWR Morb Mortal Wkly Rep* | 1994 | 1993 | **X** |  |  |  |  |  |  |  |
| CDC,OSH | Cigarette smoking among women of reproductive age--United States, 1987-1992. | *MMWR Morb Mortal Wkly Rep* | 1994 | 1987-1992 | **X** |  |  |  |  |  |  |  |
| CDC,OSH | Cigarette smoking among adults--United States, 1994. | *MMWR Morb Mortal Wkly Rep* | 1996 | 1994 | **X** |  |  |  |  |  |  |  |
| CDC,OSH | Cigarette smoking among adults--United States, 1995. | *MMWR Morb Mortal Wkly Rep* | 1997 | 1995 | **X** |  |  |  |  |  |  |  |
| CDC,OSH | Cigarette smoking among adults--United States, 1997. | *MMWR Morb Mortal Wkly Rep* | 1999 | 1997 | **X** |  |  |  |  |  |  |  |
| CDC,OSH | Cigarette smoking among adults--United States, 1998. | *MMWR Morb Mortal Wkly Rep* | 2000 | 1998 | **X** |  |  |  |  |  |  |  |
| CDC,OSH | Cigarette smoking among adults--United States, 1999. | *MMWR Morb Mortal Wkly Rep* | 2001 | 1999 | **X** |  |  |  |  |  |  |  |
| CDC,OSH | Cigarette smoking among adults--United States, 2000. | *MMWR Morb Mortal Wkly Rep* | 2002 | 2000 | **X** |  |  |  |  |  |  |  |
| CDC,OSH | Examinations for oral cancer--United States, 1992. | *MMWR Morb Mortal Wkly Rep* | 1994 | 1992 |  |  |  |  | **X** |  |  |  |
| CDC,OSH | Discomfort from environmental tobacco smoke among employees at worksites with minimal smoking restrictions--United States, 1988. | *MMWR Morb Mortal Wkly Rep* | 1992 | 1988 |  |  |  |  |  |  | **X** |  |
| CDC,OSH | Physician and other health-care professional counseling of smokers to quit--United States, 1991. | *MMWR Morb Mortal Wkly Rep* | 1993 | 1991 |  |  |  | **X** |  |  |  |  |
| CDC,OSH | Response to increases in cigarette prices by race/ethnicity, income, and age groups--United States, 1976-1993. | *MMWR Morb Mortal Wkly Rep* | 1998 | 1976-1993 | **X** |  |  | **X** |  |  |  |  |
| CDC,OSH | Smoking-attributable mortality and years of potential life lost--United States, 1988. | *MMWR Morb Mortal Wkly Rep* | 1991 | 1974-1987 | **X** |  |  |  |  | **X** |  |  |
| CDC,OSH | Smoking cessation during previous year among adults--United States, 1990 and 1991. | *MMWR Morb Mortal Wkly Rep* | 1993 | 1990, 1991 | **X** |  |  | **X** |  |  |  |  |
| CDC,OSH | Use of smokeless tobacco among adults--United States, 1991. | *MMWR Morb Mortal Wkly Rep* | 1993 | 1991 | **X** |  |  |  |  |  |  | **X** |
| Clark | Age and stage of readiness for smoking cessation. | *J Gerontol B Psychol Sci Soc Sci* | 1997 | 1992 |  |  |  | **X** |  |  |  |  |
| Cokkinides | Racial and ethnic disparities in smoking-cessation interventions: analysis of the 2005 NHIS. | *Am J Prev Med* | 2008 | 2005 |  |  |  | **X** |  |  |  |  |
| Cullen | Gender differences in chronic disease risk behaviors through the transition out of high school. | *Am J Prev Med* | 1999 | 1992 | **X** |  | **X** |  |  |  |  | **X** |
| Davis | Common courtesy' and the elimination of passive smoking. Results of the 1987 National Health Interview Survey. | *JAMA* | 1990 | 1987 |  |  |  |  |  |  | **X** |  |
| Douglas | The hazard of starting smoking: estimates from a split population duration model. | *J Health Econ* | 1994 | 1978, 1979 |  |  | **X** |  |  |  |  |  |
| Duelberg | Preventive health behavior among black and white women in urban and rural areas. | *Soc Sci Med* | 1992 | 1985 | **X** |  | **X** |  |  |  |  |  |
| Escobedo | Sociodemographic characteristics of cigarette smoking initiation in the United States. Implications for smoking prevention policy. | *JAMA* | 1990 | 1987 |  |  | **X** |  |  |  |  |  |
| Faulkner | Race and cigarette smoking among United States adolescents: the role of lifestyle behaviors and demographic factors. | *Pediatrics* | 1998 | 1992 |  |  | **X** |  |  |  |  |  |
| Geronimus | Age patterns of smoking in US black and white women of childbearing age. | *Am J Public Health* | 1993 | 1987 | **X** |  | **X** | **X** |  |  |  |  |
| Giebel | Differences in the age of smoking initiation between blacks and whites--United States. | *MMWR Morb Mortal Wkly Rep* | 1991 | 1987, 1988 |  |  | **X** |  |  |  |  |  |
| Gillum | Prevalence of cardiovascular and pulmonary diseases and risk factors by region and urbanization in the United States. | *J Natl Med Assoc* | 1994 | 1983-1987 |  |  |  |  |  | **X** |  |  |
| Gilpin | Demographic differences in patterns in the incidence of smoking cessation: United States 1950-1990. | *Ann Epidemiol* | 2002 | 1965-1992 |  |  |  | **X** |  |  |  |  |
| Gilpin | Smoking initiation rates in adults and minors: United States, 1944-1988. | *Am J Epidemiol* | 1994 | 1970, 1978-1980, 1987, 1988 | **X** | **X** | **X** |  |  |  |  |  |
| Giovino | Surveillance for selected tobacco-use behaviors--United States, 1990-1994. | *MMWR Morb Mortal Wkly Rep* | 1994 | 1965-1991 |  |  |  |  |  |  |  | **X** |
| Graubard | Analyzing health surveys for cancer-related objectives. | *J Natl Cancer Inst* | 1999 | 1987 |  |  |  |  |  |  |  | **X** |
| Hajat | Health outcomes among Hispanic subgroups: data from the National Health Interview Survey, 1992-95. | *Adv Data* | 2000 | 1992-1995 | **X** |  |  |  |  |  |  |  |
| Halpern | Differences in former smokers' beliefs and health status following smoking cessation. | *Am J Prev Med* | 1994 | 1990 |  | **X** |  |  |  |  |  |  |
| Hill | Smoking in the home and children’s health | *Tob Control* | 2008 | 1999, 2001 |  |  |  |  |  |  | **X** |  |
| Hofer | Healthy behaviors among women in the United States and Ontario: the effect on use of preventive care. | *Am J Public Health* | 1996 | 1990 |  |  |  |  | **X** |  |  |  |
| Hogan | Functional limitations and key indicators of well-being in children with disability. | *Arch Pediatr Adolesc Med* | 2000 | 19,941,995 |  |  |  |  |  |  | **X** |  |
| Horowitz | Factors associated with having oral cancer examinations among US adults 40 years of age or older. | *J Public Health Dent* | 1996 | 1992 |  |  |  |  | **X** |  |  | **X** |
| Horowitz | The need for health promotion in oral cancer prevention and early detection. | *J Public Health Dent* | 1996 | 1990, 1992 |  | **X** |  |  |  |  |  |  |
| Horowitz | U.S. adult knowledge of risk factors and signs of oral cancers: 1990. | *J Am Dent Assoc* | 1995 | 1990, 1992 |  | **X** | **X** |  |  |  |  |  |
| Husten | Cigarette smoking and smoking cessation among older adults: United States, 1965-94. | *Tob Control* | 1997 | 1965-1994 | **X** |  |  | **X** |  |  |  |  |
| Husten | Trends and effects of cigarette smoking among girls and women in the United States, 1965-1993. | *J Am Med Womens Assoc* | 1996 | 1965-1993 | **X** |  |  |  |  |  |  |  |
| Jepson | Black-white differences in cancer prevention knowledge and behavior. | *Am J Public Health* | 1991 | 1997 | **X** |  |  |  |  |  |  |  |
| Jones | Living arrangements, knowledge of health risks, and stress as determinants of health-risk behavior among college students. | *J Am Coll Health* | 1992 | 1985 |  | **X** |  |  |  |  |  |  |
| Kamimoto | Surveillance for five health risks among older adults--United States, 1993-1997. | *MMWR Morb Mortal Wkly Rep* | 1999 | 1993-1995 | **X** |  |  |  |  |  |  |  |
| King | Cigarette smoking among native and foreign-born African Americans. | *Ann Epidemiol* | 1999 | 1990-1994 | **X** |  | **X** |  |  |  |  |  |
| King | Regional variation in smoking among African Americans. | *Prev Med* | 1999 | 1990-1994 | **X** |  | **X** |  |  |  |  |  |
| King | Smoking behavior among French and American women. | *Prev Med* | 1998 | 1992, 1993 | **X** |  |  |  |  |  |  |  |
| King | Social heterogeneity in smoking among African Americans. | *Am J Public Health* | 1998 | 1990-1993 | **X** |  | **X** |  |  |  |  |  |
| Klevens | The association between veteran status and cigarette-smoking behaviors. | *Am J Prev Med* | 1995 | 1987, 1988 | **X** |  |  |  |  |  |  |  |
| Kozlowski | Most smokeless tobacco use is not a causal gateway to cigarettes: using order of product use to evaluate causation in a national US sample. | *Addiction* | 2003 | 1987 |  |  |  |  |  |  |  | **X** |
| Kuo | Health status of Asian Americans: United States, 1992-94. | *Adv Data* | 1998 | 1992-1994 | **X** |  |  |  |  |  |  |  |
| Lee | Changes in the patterns of initiation of cigarette smoking in the United States: 1950, 1965 and 1980. | *Cancer Epidemiol Biomarkers Prev* | 1993 | 1970, 1978-1980, 1987, 1988 |  |  | **X** |  |  |  |  |  |
| Levy | Employer-sponsored insurance coverage of smoking cessation treatments. | *Am J Manag Care* | 2006 | 1997-2004 |  |  |  | **X** |  |  |  |  |
| Lowry | The effect of socioeconomic status on chronic disease risk behaviors among US adolescents. | *JAMA* | 1996 | 1992 | **X** |  | **X** |  |  |  |  |  |
| Mannino | Environmental tobacco smoke exposure and health effects in children: results from the 1991 National Health Interview Survey. | *Tob Control* | 1996 | 1991 |  |  |  |  |  |  | **X** |  |
| Mannino | Environmental tobacco smoke exposure in the home and worksite and health effects in adults: results from the 1991 National Health Interview Survey. | *Tob Control* | 1997 | 1991 |  |  |  |  |  |  | **X** |  |
| Marcus | Smoking behavior among US Latinos: an emerging challenge for public health. | *Am J Public Health* | 1985 | 1979, 1980 | **X** |  |  |  |  | **X** |  |  |
| Martin | Cancer prevention in the dental practice: oral cancer screening and tobacco cessation advice. | *J Public Health Dent* | 1996 | 1992 |  |  |  | **X** |  |  |  | **X** |
| McGrady | Do sex and ethnic differences in smoking initiation mask similarities in cessation behavior? | *Am J Public Health* | 2002 | 1987 |  |  | **X** | **X** |  |  |  |  |
| Mendez | Has smoking cessation ceased? Expected trends in the prevalence of smoking in the United States. | *Am J Epidemiol* | 1998 | 1965-1993 | **X** |  |  | **X** |  |  |  |  |
| Mendez | Smoking prevalence in 2010: why the healthy people goal is unattainable. | *Am J Public Health* | 2000 | 1998 |  |  |  | **X** |  |  |  |  |
| Miller | Smoking-attributable medical care costs in the USA. | *Soc Sci Med* | 1999 | 1987 |  |  |  |  | **X** | **X** |  |  |
| Moon-Howard | African American women and smoking: starting later. | *Am J Public Health* | 2003 | 2000 |  |  | **X** |  |  |  |  |  |
| Nelson | Cigarette smoking prevalence by occupation in the United States. A comparison between 1978 to 1980 and 1987 to 1990. | *J Occup Med* | 1994 | 1978-1980, 1987-1990 | **X** |  |  |  |  |  |  |  |
| Nelson | Long-term trends in adolescent and young adult smoking in the United States: metapatterns and implications. | *Am J Public Health* | 2008 | 1976-2005,  1974-2005 | **X** |  |  |  |  |  |  |  |
| Nelson | Pipe smoking in the United States, 1965-1991: prevalence and attributable mortality. | *Prev Med* | 1996 | 1965, 1966, 1970, 1987, 1991 | **X** |  |  |  |  |  |  |  |
| Nelson | Trends in cigarette smoking among US adolescents, 1974 through 1991. | *Am J Public Health* | 1995 | 1974-1991 | **X** |  |  |  |  |  |  |  |
| Novotny | Smoking by blacks and whites: socioeconomic and demographic differences. | *Am J Public Health* | 1988 | 1985 | **X** |  | **X** | **X** |  |  |  |  |
| Novotny | Trends in smoking by age and sex, United States, 1974-1987: the implications for disease impact. | *Prev Med* | 1990 | 1974-1987 | **X** |  |  |  |  |  |  |  |
| Oster | The benefits and risks of over-the-counter availability of nicotine polacrilex ("nicotine gum"). | *Med Care* | 1996 | 1991 |  |  |  | **X** |  |  |  |  |
| Ostro | Estimating the risks of smoking, air pollution, and passive smoke on acute respiratory conditions. | *Risk Anal* | 1989 | 1976-1980 |  |  |  |  |  |  | **X** |  |
| Pierce | How long will today's new adolescent smoker be addicted to cigarettes? | *Am J Public Health* | 1996 | 1965-1988 | **X** |  | **X** | **X** |  |  |  |  |
| Pierce | News media coverage of smoking and health is associated with changes in population rates of smoking cessation but not initiation. | *Tob Control* | 2001 | 1965-1991 |  |  |  | **X** |  |  |  |  |
| Pierce | Smoking initiation in the United States: a role for worksite and college smoking bans. | *J Natl Cancer Inst* | 1991 | 1978-1980, 1987 |  |  | **X** |  |  |  |  |  |
| Pierce | Trends in cigarette smoking in the United States. Projections to the year 2000. | *JAMA* | 1989 | 1974-1985 | **X** |  |  |  |  |  |  |  |
| Pleis | Summary health statistics for U.S. adults: National Health Interview Survey, 1998. | *Vital Health Stat 10* | 2002 | 1998 | **X** |  |  |  |  |  |  |  |
| Rakowski | Smoking and cancer screening for women ages 42-75: associations in the 1990-1994 National Health Interview Surveys. | *Prev Med* | 1999 | 1990-1994 |  |  |  |  | **X** |  |  |  |
| Rodu | Switching to smokeless tobacco as a smoking cessation method: evidence from the 2000 NHIS. | *Harm Reduction Jour* | 2008 | 2000 |  |  |  | **X** |  |  |  |  |
| Rogers | Demographic and socioeconomic links to cigarette smoking. | *Soc Biol* | 1995 | 1990 | **X** |  |  |  |  |  |  |  |
| Rogers | Demographic, socioeconomic, and behavioral factors affecting ethnic mortality by cause. | *Soc Forces* | 1996 | 1986-1991 |  |  |  |  |  | **X** |  |  |
| Rogers | Ethnic differences in smoking patterns: findings from NHIS. | *Public Health Rep* | 1988 | 1979, 1980 | **X** |  |  |  |  |  |  |  |
| Rogers | Life expectancies of cigarette smokers and nonsmokers in the United States. | *Soc Sci Med* | 1991 | 1985 |  |  |  |  |  | **X** |  |  |
| Rosenbaum | Linear extrapolation models of lung cancer risk associated with exposure to environmental tobacco smoke. | *Regul Toxicol Pharmacol* | 1998 | 1987, 1992 |  |  |  |  |  | **X** | **X** |  |
| Ruchlin | An analysis of smoking patterns among older adults. | *Med Care* | 1999 | 1990 | **X** |  |  | **X** |  |  |  |  |
| Schoenborn | Health promotion in the United States and Canada: smoking, exercise, and other health-related behaviors. | *Am J Public Health* | 1988 | 1985 | **X** |  | **X** |  |  |  |  |  |
| Schuster | Smoking patterns of household members and visitors in homes with children in the United States. | *Arch Pediatr Adolesc Med* | 2002 | 1994 |  |  |  |  |  |  | **X** |  |
| Shopland | Toward the 1990 objectives for smoking: measuring the progress with 1985 NHIS data. | *Public Health Rep* | 1987 | 1985 | **X** |  |  |  |  |  |  |  |
| Siegel | Trends in adult cigarette smoking in California compared with the rest of the United States, 1978-1994. | *Am J Public Health* | 2000 | 1978-1994 | **X** |  | **X** |  |  |  |  |  |
| Sterling | An alternative explanation for the apparent elevated relative mortality and morbidity risks associated with exposure to environmental tobacco smoke. | *J Clin Epidemiol* | 1996 | 1970, 1991 |  |  |  |  |  |  | **X** |  |
| Sterling | Analysis of the relationship between smokeless tobacco and cancer based on data from the National Mortality Followback Survey. | *J Clin Epidemiol* | 1992 | 1987 |  |  |  |  |  | **X** |  | **X** |
| Sterling | Comparison of smoking-related risk factors among black and white males. | *Am J Ind Med* | 1989 | 1970, 1979, 1980 |  |  |  |  |  | **X** |  |  |
| Sterling | Smoking characteristics by type of employment. | *J Occup Med* | 1976 | 1970 | **X** |  | **X** |  |  |  |  |  |
| Sterling | The confounding of occupation and smoking and its consequences. | *Soc Sci Med* | 1990 | 1970 |  |  |  |  |  | **X** |  |  |
| Tomar | Snuff use and smoking in U.S. men: implications for harm reduction. | *Am J Prev Med* | 2002 | 1998 |  |  |  | **X** |  |  |  | **X** |
| Waldman | Just say no! sounds right, but… | *ASDC J Dent Child* | 1996 | 1992 | **X** |  |  |  |  |  |  |  |
| Weinkam | Age related changes in age of starting to smoke. | *J Clin Epidemiol* | 1990 | 1970, 1979, 1980 |  |  | **X** |  |  |  |  |  |
| Weinkam | Smoking and hospital utilization. | *Soc Sci Med* | 1987 | 1970 |  |  |  |  | **X** |  |  |  |
| Wetzler | Self-reported physical health practices and health care utilization: findings from the National Health Interview Survey. | *Am J Public Health* | 1985 | 1977 | **X** |  |  |  | **X** |  |  |  |
| Willard | Relationship between cigarette smoking and other unhealthy behaviors among our nation's youth: United States, 1992. | *Adv Data* | 1995 | 1992 |  |  |  |  | **X** | **X** |  |  |
| Young | Health promoting behaviors of family practice residents: do they compare with the general public? | *Fam Med* | 1988 | 1985 | **X** |  |  | **X** |  |  |  |  |
| Zhu | The relationship between cigarette smoking and education revisited: implications for categorizing persons' educational status. | *Am J Public Health* | 1996 | 1983-1991 | **X** |  |  |  |  |  |  |  |
