# Supplementary material for: Using the National Health Interview Survey to understand and address the impact of tobacco in the United States: past perspectives and future considerations
Source: Epidemiol Perspect Innov. 2008 Dec 4;5:8. doi: 10.1186/1742-5573-5-8 (PMC2627846; doi:10.1186/1742-5573-5-8)
Supplement: Additional file 4 — Analysis of NHIS Data: Prevalence and Trends. [file 1742-5573-5-8-S4.doc]

# Table 4. Analysis of NHIS Data: Prevalence and Trends

| **Specific Population** | **Data Source** | **Research Question** | **Reported Findings** | **Reference** |
| --- | --- | --- | --- | --- |
| **All Forms of Tobacco** | | | | |
| **Adults** | 2005 NHIS | What is the progress toward achieving the 2010 national health objective to reduce the prevalence of cigarette smoking to 12%, cigar smoking to 1.2%, and smokeless tobacco to 0.4%, and to increase cessation attempts to 75%? | Approximately 20.9% of adults were current cigarette smokers, the same percentage as in 2004, suggesting a stall in the 8-year decline in smoking prevalence among adults in the United States. In addition, an estimated 2.2% of adults were current cigar smokers, 2.3% used smokeless tobacco, and 42.5% of current cigarette smokers had stopped smoking for at least 1 day in the preceding 12 months because they were trying to quit. | CDC, 2006; 42;1145 |
|  | 1987 CEC | What are smoking and other tobacco-use practices among a representative sample of adults in U.S. households? | Approximately 33% of U.S. adults regularly used some form of tobacco. Use varied by gender, age, race/ethnicity, and marital status. | Boyd, 1989 |
| **Cigarettes** | | | | |
| **Adolescents and Young Adults** | MTF, 1976-2005 (adolescents); NHIS, 1974-2005 (young adults) | What are the long-term trends in adolescent and young adult smoking in the United States? | Analysis revealed a large increase and subsequent decrease in overall smoking over the 15 years of study; a steep decline in smoking among Blacks through the early 1990s; a gender gap reversal among older adolescents and young adults; similar trends in smoking for most subgroups since the early 1990s; and a large decline in smoking among young adults with less than a high school education | Nelson et al., 2008 |
| **Adults** | 2000 NHIS SAMPLE ACQ, CCM | What is the progress toward achieving the 2010 national health objective to reduce the prevalence of cigarette smoking among adults to ≤ 12%? | Approximately 23.3% of adults were current smokers, compared with 25.0% in 1993, a modest but statistically significant decrease in smoking prevalence among U.S. adults. Approximately 70% of smokers reported wanting to quit in 2000; 41% had tried to quit during the preceding year. Quitting success varied among demographic groups. | CDC, 2002; 29;642 |
|  | 1985 HPDP | What is the progress toward achieving the four 1990 national health objectives concerning cigarette smoking? | The objective of reducing the proportion of adults who smoke to less than 25% had not been achieved. In general, the three objectives concerning the population’s awareness of the health consequences of cigarette smoking had been met. | Shopland & Brown, 1987 |
|  | 1974-87 NHIS | What are the smoking attributable mortality (SAM), years of potential life lost (YPLL), and age-adjusted SAM and YPLL rates for the United States in relation to smoking prevalence? | In 1988, approximately 434,000 deaths and 1,199,000 YPLL before age 65 (6,028,000 before age 85) were attributable to cigarette smoking. Although SAM for Blacks represented 11% of total SAM, the SAM rate for Blacks was 12% higher than for Whites. The SAM for men was 66% of total SAM, and the SAM rate for men was more than twice the rate for women. The rate of smoking-attributable YPLL before age 65 for Blacks was twice that for Whites, and the smoking-attributable YPLL rate for men was almost three times that for women. For YPLL before age 85, the rate for Blacks was 52% higher than for Whites; YPLL for men was more than twice that for women. | Schultz, 1991 |
|  | 1978-80 NHIS Smoking | What are the prevalence and levels of cigarette smoking among groups of U.S. workers? | The percentage of current smokers was higher among men than women in the general population, but few differences were found in the prevalence of smoking among men and women for specific occupations. Race and employment status influenced smoking prevalence, as did industry. | Brackbill, Frazier, & Shilling, 1988 |
|  | 1998 NHIS SAMPLE ACQ | What progress has been made toward meeting the 2010 national health objective of reducing prevalence of cigarette smoking among adults to 12%? | In 1998, 24.1% of adults were current cigarette smokers (26.4% male and 22% female) and 22.9% were former smokers. Of the current smokers, 19.7% reported everyday smoking, and 4.2% reported smoking on some days. Current smoking prevalence was highest among persons ages 18-24 (27.9%) and ages 25-44 (27.5%), and lowest among persons ages ≥65 years (10.9%). Prevalence was highest among American Indians/Alaska Natives (40%), intermediate among non-Hispanic Whites (25%) and Blacks (24.7%), and lowest among Hispanics (19.1%) and Asian/Pacific Islanders (13.7%). Only adults with ≥16 years of education met the 2010 goal, with prevalence at 11.3%. Persons with 9-11 years of education had a smoking prevalence of 36.8%, and persons living below the poverty level had a prevalence of 32.3%. An estimated 39.2% of current smokers had made a quit attempt during the past 12 months. | CDC, 2000; 49:881 |
|  | 1978-80, 1983, 1985, 1987, 1988, 1990-94 BRFSS | How do trends in adult cigarette smoking prevalence compare in California and the remainder of the United States between 1978 and 1994? | Overall, the estimated annual rate of decline in adult smoking prevalence accelerated significantly between 1985-90 and slowed significantly from 1990-94. In contrast, California attained better results than the rest of the United States due to more aggressive tobacco control intervention. | Siegel et al., 2000 |
|  | 1983, 1985, 1987, 1988, 1990-91 NHIS | What is the relationship between education and smoking? | Smoking rate peaks for those with 10-11 years of education and then declines steeply and steadily. | Zhu et al., 1996 |
|  | 1999 NHIS SAMPLE ACQ | What progress has been made toward meeting the 2010 national health objective of reducing prevalence of cigarette smoking among adults to 12%? | 1999 survey results indicated that 23.5% of adults were current smokers (25.7% male and 21.5% female) and 23.1% were former smokers. Of the current smokers, 19.2% reported everyday smoking, and 4.3% reported smoking on some days. Current smoking prevalence was highest among persons ages 18-24 (27.9%) and ages 25-44 (27.3%) and lowest among persons ages ≥65 (10.6%). Prevalence was highest among American Indians/Alaska Natives (40.8%) and lowest among Hispanics (18.1%) and Asian/Pacific Islanders (15.1%). Only adults with masters, doctoral, or professional degrees met the 2010 goal with prevalence at 8.5%. Persons who earned a GED had a smoking prevalence of 44.4%, and persons living below the poverty level had a prevalence of 33.1%. An estimated 41.3% of current smokers had made a quit attempt during the past 12 months. | CDC, 2001; 40;869 |
|  | 1990 HPDP | What is the prevalence of cigarette smoking among adults in the United States? | The 1990 survey results indicated that 50.1% of adults were ever smokers, with 25.5% of adults reporting current smoking (28.4% male and 22.8% female). Current smoking prevalence was highest among persons ages 25-44, American Indians/Alaska Natives, non-Hispanics, and persons with <12 years of education.  In 1990, 26.4% of respondents ages 20-24 years reported current smoking (28.6% male and 24.3% female). In this age group, 28.3% of Whites and 17.3% of Blacks reported smoking. | CDC, 1992; 20; 354 |
|  | 1992 CCES | What is the prevalence of adult smoking and the impact of change in definition used to assess self-reported smoking prevalence (inclusion of intermittent smokers)? | The overall prevalence estimate from 1991 to 1992 did not increase. However, when intermittent smokers were included in 1992, the prevalence estimate increased from 25.6% in 1991 to 26.5% in 1992. In 1992, prevalence of daily smoking was 22.1% and prevalence of some-day (intermittent) smoking was 4.4%. Smoking prevalence was highest among the 25-44 age group (30.8%); American Indians/Alaska Natives (39.4%); and persons living below the poverty level (34.9%). Prevalence was lowest among Asian/Pacific Islanders (15.2%) and declined with increasing levels of education. Prevalence was higher among men (28.6%) than women (24.6%). | CDC, 1994; 19; 342 |
|  | 1993, NHIS-2000 Obj Suppl | What is the prevalence of smoker desire to quit and prevalence of cessation? | Prevalence of daily smoking in 1993 was significantly lower than in 1992; 69.7% wanted to quit smoking completely. Women were more interested in quitting than men (72.7% vs. 67.1%), while smokers ages 65+ were least likely to give up. | CDC, 1994; 50;925 |
|  | 1994, NHIS-2000 Obj Suppl | What progress has been made toward the year 2000 smoking reduction objective? | Overall prevalence of smoking for sociodemo­gra­phic subgroups was unchanged from 1993 to 1994. Except for persons with 0-8 years of education, prevalence varied inversely with education level and was highest among persons with 9-11 years of schooling (38.2%). | CDC, 1996; 27; 588 |
|  | 1965-93 NHIS  1986 NMFS | Can the future prevalence of smoking be predicted? | Cessation rates have been reasonably stable over the last 15 years. Prevalence issues are more likely to be due to initiation rates vs. cessation rates. Although prevalence of smoking among adults will continue to decline even with initiation rates rising to 35%, eventual decline will be modest due to 27% initiation rate by youth. | Mendez et al., 1998 |
|  | 1974, 1976-80, 1983, 1985, 1987 NHIS | What are the trends in current smoking prevalence and quit ratios by age/sex and the implications for disease impact? | Smoking prevalence decreased linearly for men in all age subgroups except those ages 65+, and for women ages 25-44 and 45-64 only. Quit ratios increased except for ages 20-24. Overall, there were 1.4 million fewer women smokers and 1 million more men smokers in 1987 than in 1974; the actual number of smokers increased among men ages 25-44 (8% increase), women ages 25-44 (15% increase), and those ages 65+ (50% increase). | Novotny et al., 1990 |
|  | 2003 NHIS | What progress has been made toward achieving the objective to reduce the prevalence of cigarette smoking among adults to 12% by 2010? | In 2003, 21.6% were current smokers: 81.0% smoked daily, 19.0% smoked some days, 41.1% tried to quit. During 1983-2003, there was a sustained smoking decline in all groups except those ages 18-24; in this group, prevalence increased during 1993-2002 to 28.5% and went down to 23.9% in 2003, the lowest since 1991. | CDC, 2005; 20; 509 |
|  | 2004 NHIS | What progress has been made toward achieving the objective to reduce the prevalence of cigarette smoking among adults to 12% by 2010? | In 2004, among 20.9% current smokers, 81.3% smoked daily, 18.7% smoked some days, and 40.5% tried quitting. From 1993 to 2004, daily smokers of 25 cigarettes per day decreased from 19.1% to 12.1%, while someday smokers remained at 18-19%. | CDC, 2005; 44; 1121 |
|  | 2005 NHIS | What is the health status of U.S. adults in relation to smoking? | 21% current cigarette smokers (23% men, 18% women), 21% former smokers, 58% had never smoked (51% men, 64% women). Current smokers: 13% Asian, 21% White, 21% Black, 25% American Indian/Alaska Native. Never smokers: 54% non-Hispanic White, 64% non-Hispanic Black, 69% Hispanic. Those less likely to be current smokers: with a bachelor’s degree or more; not poor; did not live in an MSA; living in the West. Current women smokers: 11% Hispanic, 17% non-Hispanic Black, 21% non-Hispanic White. Women nonsmokers: 79% Hispanic, 71% non-Hispanic Black, 59% non-Hispanic White. Current men smokers: 20% Hispanic, 26% non-Hispanic Black, 24% non-Hispanic White. Current men non-smokers: 59% Hispanic, 56% non-Hispanic Black, 49% non-Hispanic White. | Pleis & Lethbridge-Cejku, 2006 |
|  | 1987-94, 1997-2004 NHIS | Does the gap in smoking rates between blue- and white-collar workers over the past four decades continue into the new millennium? | For each occupational group, the pooled smoking rates were lower in the 1997-2004 survey period relative to years 1987-1994. In the most recent survey period, the pooled rate for all workers was 24.5%, from the highest 38.8% of construction workers to the lowest 5.0% in the health diagnosing professions. There were significant annual reductions in smoking rates for all U.S. workers in both survey periods (0.6%), with a slightly lower yearly reduction over the entire survey period (0.4%). The majority of blue-collar worker groups had pooled 1997-2004 smoking rates in excess of 24.5%. | Lee et al., 2007 |
|  | 1991 HPDP | What progress has been made in reducing smoking prevalence? | In 1991, an estimated 49.8% of adults ever smoked, and 25.7% currently smoked (28.1% of men and 23.5% of women). Smoking was most prevalent among people ages 25-44 years (30.4%). Estimated smoking prevalence was highest among American Indian/Alaska Native populations (31.4%) and lowest among Asian/Pacific Islander (16%) and among Hispanic women (15.5%). Smoking prevalence among people living below the poverty level was 33.1%. Approximately 48.5% of those who ever smoked were former smokers (51.6% for men and 44.7% for women). The proportion of ever smokers who were former smokers increased with education (41.8% for <12 years, 66/1% for ≥16 years). Mean number of cigarettes smoked daily per smoker was 20 (21.6 for men and 18.3 for women). Whites smoked more than Blacks (21 vs. 15 cigarettes/day) and non-Hispanics more than Hispanics (20.4 vs. 13.4). Smokers at or above the poverty level smoked more than those below the poverty level (20.3 vs. 18.7 cigarettes/day). | CDC, 1993;12; 230 |
|  | 1977 NHIS | What is the relationship between health practices and health care utilization in a national sample in relation to smoking behavior? | The never smokers reported fewer visits to the dentist. The trends for smoking cigarettes support the association of lower utilization with better health practices. | Wetzler & Cruess, 1985 |
|  | 1995 Year 2000 Objectives | What progress has been made toward the year 2000 smoking reduction objective? | Overall prevalence of smoking in 1995 of 24.7% was similar to the 25.5% of 1994. The goal of 15% prevalence by 2000 would not be reached. | CDC, 1997; 51;1217 |
|  | 1997 NHIS | What progress has been made toward the national health objective of 15% adult cigarette smoking prevalence in US? | 22.8% of adults were former smokers, including 25.1 million men and 19.2 women. 24.7% of adults were current smokers and the overall prevalence of current smoking in 1997 was unchanged from the prevalence in 1995. | CDC, 1999; 43;993 |
|  | 1998 NHIS | What is the overall prevalence of smoking among U.S. adults in 1998? | Overall, nearly one-fourth of adults were current smokers, while 23% were former smokers and 53% had never smoked; 26% of men were current smokers compared with 22% of women; and 59% of women and 46% of men had never smoked. | Pleis & Coles, 2002 |
|  | 1985, 1990 HPDP | What are the trends in adult smoking habits and the relationship between smoking and occupational and income group? | Smoking rates in 1990: males, 28%; females, 23%. Between1985-90 the only demographic groups with rising rates were males 18-24 years, females ages 65 and over, those with <12 years education, and adults of Hispanic origin. By occupational category, 40% of men employed as laborers/movers were current smokers vs. 17% and 21%, respectively, of professional and executives; lowest current smoking rates were among female professionals, technical personnel, and executives. In all, in­come was inversely related to smoking status. | “Cigarette Smoking…,” *Stat Bull Metrop Insur Co*, 1992 |
|  | 1978-80 NHIS Smoking Suppl.,  1987-90 CE, CC, OHS, HPDP | What are the changes in cigarette smoking prevalence by occupation in 1978-80 compared with 1987-90? | Since 1978-1980, differences in smoking prevalence by occupation have widened, providing further evidence that smoking has moved from a relatively common behavior practiced by most segments of society to one that is more concentrated among selected subpopulations. | Nelson, Emont, et al., 1994 |
| **Female Adults** | 1965-93 NHIS | What is the trend in smoking prevalence among women? | Overall prevalence of smoking in women is declining at a rate comparable to that of men; the rate remains high in American Indians. | Husten et al., 1996 |
| **American and French**  **Females** | 1992-93  NHIS  1992-93  CFES (France) | What are the sociodemographic factors related to cigarette smoking prevalence and the number of cigarettes smoked daily among nationally representative samples of French and U.S. women? | Prevalence of smoking among French women was significantly higher than among U.S. women (30.8% vs. 26.3%) especially among younger age groups. The average number of cigarettes smoked/day was significantly lower for French women (12.3) than for U.S. women (18.2). Regression analy­sis revealed statistic­al­ly significant inter-action terms differentiating the impact of marital status, age, and education on the smoking status of all women, as well as the socio­demo­graphic determinants of the number of cigarettes smoked. Education was inversely related to smoking among U.S. women but was positively associated with the smoking behavior of French women. | King, Grizeau, et al., 1998 |
| **Black and White**  **Females** | 1987 CCS | What are the relationships between smoking and age and race? | White women initiate smoking at younger ages but are more likely to quit. | Geronimus et al., 1993 |
| **Blacks** | 1990-94 NHIS | What are the differences in current smoking status and the number of cigarettes consumed daily between foreign and native-born African Americans and the impact of demographic and socioeconomic (SES) factors on smoking behavior? | Native-born African Americans were more likely to be current smokers than foreign-born Blacks. In the native-born group, smoking prevalence decreased with increasing education and income; these associations were not found for foreign-born Blacks. Women in both groups were less likely than men to be current smokers. Statistically significant differences in the number of cigarettes smoked per day were not found between the two groups. | King, Polednak, Bendel, & Hovey, 1999 |
|  | 1990-93 NHIS;  1992  CCE | What are the effects of SES and demographic indicators on recent smoking behavior of Blacks? | Age was the strongest predictor of smoking. Men were at least 1.75 times as likely as women to be smokers. Individuals with annual incomes of <$15K had the highest smoking prevalence rates and individuals with incomes of >$50K had the lowest prevalence. Age was the single strongest predictor of smoking, followed by education. Smoking decreased with increasing education for all age and gender groups except women ages 55-64. Smoking rates for men peaked in the 35-44 year age group. Compared with the 18-24 age group, people ages 35-44 were 3.4 (in 1990) to 7 times (in 1993) more likely to smoke. The Midwest region had the highest prevalence rates. For 3 of the 4 years examined, gender was the only statistically significant predictor of the number of cigarettes smoked per day. In 1990 and 1993, men were twice as likely as women to smoke 15+ cigarettes per day. | King & Bendel, 1998 |
|  | 1987 CCS | Is race a significant factor in the relationship between cancer prevention knowledge and behavior? | When knowledge factors were included in the models, race was not a significant predictor, except that Black females smoke less than White females. | Jepson et al., 1991 |
|  | 1990-94 NHIS | What are the differences in current smoking among Black respondents? | Gender and regional factors such as social history of migration, social stress and racism, exposure to tobacco ads, variations in cultural influences, community structures, and coping strategies have an effect on Black smoking behavior. | King, Polednak, & Bendel, 1999 |
|  | 1974-85 Smoking Suppl | What will the projected trends in cigarette smoking be in the United States by 2000? | Smoking is decreasing at a steady rate. The decline in smoking prevalence has been un­equ­al across sociodemographic subpop­ula­tions. Prevention efforts have been less effective than efforts to promote cessation. | Pierce et al.,1989 |
| **Older Adults** | 1990 HPDP | What are the national point prevalence profiles and associations between age, health status, and health beliefs of older adults who ever smoked or currently smoke? | Among adults above age 54, 53% smoked in the past, 17% smoked in 1990, 61% of smokers tried to quit, and 36% noted that their physicians never advised them to quit. | Ruchlin,  1999 |
|  | 1965-94 NHIS | What are the patterns of cigarette smoking and smoking cessation among older adults in the United States? | Smoking prevalence for 65+ declined from 1965-1994 and was lower among older than younger adults. Cessation prevalence rose with educational level and was higher for men than for women and for Whites than for Blacks. No racial differences were found among women. Older White and Hispanic men were significantly more likely to be former smokers than older Black men. | Husten et al., 1997 |
| **Black, Hispanic**  **Older Adults** | 1993-95 NHIS  1994-97 BRFSS | What effects do demographic factors have on the prevalence of five major health risks among older Black and Hispanic adults? | For the 50 states, prevalence of smoking decreased with increasing age for those 55+. Sex and age were differentially associated with all five health risks. | Kamimoto et al., 1999 |
| **Mexican Americans, Blacks** | 1990 HPDP | What are the underlying demographic and SES factors related to multiple smoking statuses? | Age displays distinct, curvilinear patterns with smoking; men have higher rates of cigarette consumption; Whites, especially males, exhibit high probabilities of cigarette consumption but also of being former smokers; Black males exhibit high probability of light smoking, but only at the older ages, and high probability of being light former smokers; Mexican American women are the least likely currently to smoke or to have smoked. | Rogers et al., 1995 |
|  | 1979-80 Smoking Suppl | What are smokers’ characteristics within ethnic groups? | Ethnic differences in smoking vary across several dimensions. Many Mexican American women never smoke. If they do, they began smoking late, smoke few cigarettes per day, and are likely to quit. Although many White men and women have tried smoking, a much higher proportions of men have quit. Blacks generally display rates intermediate to Mexican Americans and Whites, with relatively low rates of cigarette consumption. | Rogers & Crank, 1988 |
| **Hispanics** | 1992-95 AIDS  1992 CES  1993-95 Y2K | What differences in smoking patterns can be found among Hispanic subgroups? | Little difference was seen among men in terms of smoking status. From age-adjusted data, there were more current smokers among Puerto Rican women (20.7%) than Cuban (11.6%), Mexican American (12.2%), and “other Hispanic” (14.7%) women. | Hajat et al., 2000 |
|  | 1979-80 NHIS & State and city-specific surveys | What is the evidence about smoking and lung cancer among Latinos? | A notable sex difference in smoking among Hispanics was found, with male smoking rates as high or greater than that of White men, while Hispanic women report rates consider­ably lower. The pattern of smoking rates among men and women generalizes across Hispanic subgroups, including country of origin. | Marcus & Crane, 1985 |
|  | 1997-2001 NHIS | What are the ethnic variations in health among Hispanic adults and can they be explained? | NHIS surveys reveal health patterns are clearly different among Hispanic ethnic groups. They are less likely than Whites to incur health risks due to smoking. | Zsembik & Fennell, 2005 |
| **Black, Hispanic**  **Women** | 1990-91 HPDP | Has smoking prevalence among Blacks and Hispanics declined? | Prevalence of smoking among Black and Hispanic women declined 3.7% from 1987 to 1992. | CDC, 1993; 26;504 |
| **Minority, Low SES, All Ages** | 1976-80, 1983, 1985, 1987-1993 | What is the potential impact of cigarette price increases on minority and lower income populations? | Lower income, minority, and younger populations would be more likely to reduce or quit smoking in response to cigarette price increases. | CDC, 1998; 29; 605 |
| **Blacks, Whites** | 1985 NHIS | What are the independent effects of race, SES, and demographic factors on ever smoking, quitting, and heavy smoking? | The odds of ever smoking are not higher for Blacks compared with Whites when other variables are controlled. By contrast, the odds of heavy smoking for Blacks are far less than for Whites, while Blacks are significantly less likely than Whites to quit smoking, regardless of SES or demographic factors. | Novotny et al., 1988 |
|  | 1970 NHIS | Are there pronounced differences in prevalence intensity, kind, and cessation of smoking related to type of employment? | There are pronounced differences in prevalence intensity, kind, and cessation of smoking related to type of employment. Sex, race, and occupation reflect physiological, social, cultural, and economic conditions that influence the prevalence and amount of cigarette smoking. | Sterling, 1976 |
| **Black, White**  **Females** | 1985 NHIS | What is the relationship of race and health behavior for Black and White women? What possible differences exist between rural and urban residents concerning their health behavior? | Black women are less likely to engage in primary prevent­ion behaviors yet are more likely to engage in secondary ones. The higher percentage of smoking among them is due to their lower levels of education. Urban Black women are most likely to be smokers. | Duelberg, 1992 |
| **Whites** | 1965-88 NHIS | What are the trends in cessation patterns and can they be used to project future experience? | The median cessation age for those who started smoking as adolescents is expected to be 33 for men and 37 for women; 50% of these adolescents may smoke for at least 20 years, based on a median initiation age of 16-17. Despite a decline in median age of smokers who quit, smoking will be a long-term addiction for many adolescents who start now. | Pierce & Gilpin, 1996 |
| **White, Black, Hispanic**  **Adolescents** | 1992 YRBS | What is the prevalence of smoking among adolescents and young adults by race and age? | Three-fourths of those ages 18-21 had tried or currently smoke cigarettes. Among children ages 12-13, 13% tried smoking and 8% are current smokers; among them 17% boys and 9% girls smoked daily in the past month. There was greater prevalence among White males and females and male Hispanics than among Blacks. Among Whites, 13% first smoked regularly before age 13. More than two-thirds of 12- to 13-year-old smokers have tried to quit smoking, compared with 57% of 14- to 17-year-old smokers and one-half of 18- to 21-year-old smokers. | Waldman*,* 1996 |
| **Black, Hispanic**  **Adolescents** | 1992 YRBS | What is the prevalence of cancer risk behaviors related to SES status among Black and Hispanic adolescents? | Among Black and Hispanic adolescents, 63% reported two or more risk behaviors; as income level increased, they were less likely to smoke. | Lowry et al.,1996 |
| **Black**  **Adolescents** | 1974, 1976, 1978- 80, 1983, 1985, 1987-88, 1990-91 NHIS | How did adolescent smoking change from 1974 to 1991? | Overall smoking prevalence declined much more rapidly between 1970 and 1984 than from 1985 to 1995. | Nelson et al., 1995 |
| **Black**  **Veterans** | 1987 CCS  1988 OHS | What is the prevalence of smoking among veterans? | Veterans were more likely to have smoked than nonveterans in all age categories. | Klevens et al., 1995 |
| **Health Care Providers** | 1974, 1976-80, 1983, 1985, 1987-88, 1990- 91 NHIS | What are the trends in smoking prevalence among physicians, RNs, and LPNs? | Physicians are far less likely to smoke than the general public; RNs smoke less than the general population since the mid-1970s; LPNs’ prevalence is higher than the general population. | Nelson, Giovino, et al., 1994 |
| **Family Medicine Residents** | 1985 NHIS  1985 Family Medicine  Resident Survey | How do the health-promoting behaviors of family medicine residents compare with those of the general population? | Less than 5% of the family medicine residents were currently smoking vs. 28% in NHIS; 86.4% of female resi­dents and 77.4% of male residents had never smoked, compared with their respective counterparts’ rates of 55.4% and 45.3%. Residents were more likely to counsel patients about such health risks if they were nonsmokers and very moderate drinkers. Residents were modeling health-promoting behaviors at a significantly higher rate than their national counterparts. | Young, 1988 |
| **Canadians vs. Americans** | 1985 HPDP,  1985 Canada Health Promotion Study | What differences in health behaviors exist between the United States and Canada? | Canadians smoke more than U.S. adults. | Schoenborn & Stephens, 1988 |
| **Ages 10-34** | 1970, 1978-80, 1987-88 NHIS | What are the trends in tobacco use among adolescents and adults? | Reductions in tobacco use occurred in all subgroups except White male high school seniors (smoking) and White males ages 18-34 (smokeless). | Gilpin et al.,1994 |
| **Asian Americans** | 1992-94 Spec Health Topics | What is the health status of men in selected Asian national origin groups? | Among Asian/Pacific Islanders, 22.3% of men and 8.2% of women were current smokers. Among current smokers, 8.7% were Asian Indians, 10% were Chinese, 17.4% were Filipino, 19.4% were Japanese, 22.5% were Korean, and 13.2% were Vietnamese. Rates among male current smokers: Filipino, 27%; Japanese, 26%; Korean, 23%; and Chinese, 16%. | Kuo & Porter, 1998 |
| **Asian Adults** | 2004-2006 NHIS | How do Asian adults compare among Asian subgroups and with non-Hispanic Whites, non-Hispanic Blacks, non-Hispanic American Indians/Alaska Natives (AI/AN), and Hispanics for selected health status indicators, health behaviors (including smoking), health care utilization, health conditions, immunizations, and HIV testing status | Most Asian adults had never smoked; rates ranged from 65% of Korean adults to 84% of Chinese adults. Korean adults (22%) were about 2 to 3 times as likely to be current smokers as were Japanese (12%), Asian Indian (7%), or Chinese adults (7%). AI/AN adults (32%) had the highest prevalence of current cigarette smoking and Asian adults had the lowest (12%), compared with Whites (23%), Blacks (21%), and Hispanics (15%). | Barnes, Adams, & Powell-Griner, 2008 |

| **Smokeless Tobacco** | | | | |
| --- | --- | --- | --- | --- |
| **Black, White**  **Females** | 1987-92 NHIS | How has smoking behavior among women changed between 1987 and 1992? | In 1992, it was estimated that 14.3 million U.S. women ages 18-44 smoked. Prevalence of cigarette smoking among women of reproductive age in the United States declined 3.7% from 1987 to 1992. There was a substantial decline from 1997 (29.6%) to 1990 (25.6%) but a slight increase from 1991 (26.7%) to 1992 (26.9%). Smoking prevalence was inversely related to level of education and was consistently highest among those with less than a high school education. Among women ages 18-24, smoking prevalence among Black women decreased dramatically during 1987-91, but remained unchanged among White women. | CDC, 1994; 43;789 |
| **Adults** | 1991 HPDP | What are the trends in the prevalence of use of smokeless tobacco products? | An estimated 2.9% of adults were current users (5.6% men and 0.6% women). For men, prevalence of use was highest among those ages 18-24 (8.2%). In women, prevalence was highest among those ≥75 years (2.3%). Prevalence of use was highest among American Indian/Alaska Native populations for both genders (8.1% for men, 2.5% for women), followed by Whites for men (6.2%), and Blacks for women (2.3%). Prevalence of use declined with increasing education. Prevalence of use was higher in rural areas (6%) and in the South (4.6%). Prevalence of use was higher among those living below the poverty level (3.7%), but this finding was significant only for women (p<0.05). An estimated 4.4% reported former smokeless tobacco use. | CDC, 1993; 14; 263 |
| **Cigarettes and Smokeless Tobacco** | | | | |
| **Adolescents** | 1992 YRBS | What is the prevalence of cigarette smoking and smokeless tobacco use in adolescents among a wide range of health-risk behaviors? | Lifetime cigarette use increased significantly with age group, and current use of smokeless tobacco was significantly higher among the older age groups. One fourth of all adolescents ages 12-13 engaged in at least one health-risk behavior. | CDC, 1994; 43: 231 |
|  | 1992 YRBS | What is the smoking and snuff prevalence among adolescents? | About one-half of adolescents had ever smoked a whole cigarette. The percent of those who had tried to quit declined steadily with age; use of chewing tobacco and snuff was much lower than cigarette use and was highest in White males | Adams et al., 1995 |
|  | 1992 YRBS | Do health-related behaviors change at times of major life transitions and are there differences by gender? | Significant gender differences by transition effects were obtained for snuff use; daily and heavy cigarette smoking increased during high school years. | Cullen et al., 1999 |
| **Black, Hispanic**  **Adolescents** | 1992 YRBS | What are the differences by ethnic group in the performance of cancer risk related lifestyle behaviors through the transition out of high school? | Cancer risk increased at the transition out of high school through changes in cancer-related behaviors. Hispanic males experienced somewhat higher risks for chewing tobacco and snuff use after the transition out of high school. | Baranowski, et al., 1999 |
| **Pipes and Cigarettes** | | | | |
| **Blacks**  **Under Age 20** | 1965-66, 1970, 1987, 1991 NHIS | What is the estimated attributable risk of death from pipe smoking? | Pipe smoking has declined drastically over three decades. | Nelson et al., 1996 |

* Study Population can be assumed to be adult males and females, unless otherwise stated. Categories reflect the authors’ terminology used to describe their sample and does not imply consistency among population parameters.
