# Supplementary material for: Using the National Health Interview Survey to understand and address the impact of tobacco in the United States: past perspectives and future considerations
Source: Epidemiol Perspect Innov. 2008 Dec 4;5:8. doi: 10.1186/1742-5573-5-8 (PMC2627846; doi:10.1186/1742-5573-5-8)
Supplement: Additional file 5 — Analyses of NHIS Data: Attitudes, Knowledge, and Beliefs about Tobacco Use. [file 1742-5573-5-8-S5.doc]

# Table 5. Analyses of NHIS Data: Attitudes, Knowledge, and Beliefs about Tobacco Use

| **Specific Population** | **Data Source** | **Research Question** | **Reported Findings** | **Reference** |
| --- | --- | --- | --- | --- |
| **Adults** | 1990 HPDP Supplement | How do beliefs and the self-reported health of former smokers (FS) change with time after smoking cessation? | Beliefs of FS about smoking-relat­ed health risks may strengthen with time foll­ow­ing cessation. FS are more likely than current smokers to engage in a wide range of health practices unrelated to smoking or smok­ing-induc­ed illnesses and to have greater general health knowledge. FS with the strongest be­liefs about health risks are most likely to remain abstinent. | Halpern and Warner, 1994 |
| **Ages 10-24** | 1970, 78, 79, 80, 87, 88 Smoking  Supplements | Has knowledge of health consequences of smoking led to a decrease in initiation rates? | Smoking initiation in adult males declined sharply around 1950; the decline for adult females began in the mid-1960s; rates for females 10-14 and 15-20 increased through the 1970s. | Gilpin et al., 1994 |
| **College Students,**  **Ages 18-25** | 1985 HPDP | What is the association between knowledge of health risks, living arrangements, and perceived stress with health-risk behaviors in college students? | Smoking may be less influenced by health knowledge and more associated than drinking with a response to stress. | Jones et al, 1992 |
| **Black, Hispanic**  **Low SES** | 1992 CCS | What is the knowledge of risk factors among Americans? | Most respondents were unable to identify major cancer risk factors when prompted with a list. In women, 32.1% identified smoking as a cancer risk factor for breast cancer, 12.9% for cervical cancer, and 10.6% for colon cancer. In men, 16.3% identified smoking as a cancer risk factor for prostate cancer, and 10.6% for colon cancer. | Breslow et al., 1997 |
| **Adults** | 1990 HPDP Supplement  1992 CCS | What percentage of adults know the risk factors for oral cancer and recognize the signs? | Two-thirds of respondents identified tobacco use as a risk for oral cancer. Lack of knowledge persisted across all groups analyzed. | Horowitz et al., 1995 |
| **Age 40+** | 1990 HPDP Supplement  1992 CCS | What is public and professional knowledge and use of oral cancer examinations and the rationale for cancer prevention? | 1990: U.S. adults, regardless of age, are not well informed about signs of oral cancer.  1992: 14% examined for oral cancer, two-thirds by a dentist. Least likely to have exams were Blacks or Hispanics, current tobacco users, and those age 65+, with low education, and with low knowledge of risk factors. | Horowitz, Parivash, & Nourjah 1996 |

* Specific Population can be assumed to be adult males and females, unless otherwise stated. Categories reflect the authors’ terminology used to describe their sample and does not imply consistency among population parameters.
