# Supplementary material for: Using the National Health Interview Survey to understand and address the impact of tobacco in the United States: past perspectives and future considerations
Source: Epidemiol Perspect Innov. 2008 Dec 4;5:8. doi: 10.1186/1742-5573-5-8 (PMC2627846; doi:10.1186/1742-5573-5-8)
Supplement: Additional file 6 — Analyses of NHIS Data: Initiation of Cigarette Smoking. [file 1742-5573-5-8-S6.doc]

# Table 6. Analyses of NHIS Data: Initiation of Cigarette Smoking

| **Specific Population** | **Data Source** | **Research Question** | **Reported Findings** | | **Reference** | |  | | | |
| --- | --- | --- | --- | --- | --- | --- | --- | --- | --- | --- |
| **Adults** | 1978-80, 1983, 1985, 1987-88, 1990-94 NHIS  1984-94 BRFSS | How do trends in adult cigarette smoking prevalence compare between California and the remainder of the United States between 1978 and 1994? | Overall, the estimated annual rate of decline in adult smoking prevalence accelerated significantly between 1985 and 1990 and slowed significantly from 1990 to 1994. In contrast, California attained better results than the rest of the United States due to more aggressive tobacco control intervention. | | Siegel et al., 2000 | |  | | | |
|  | Year 2000 Objectives | What are the effects of weight control concerns on smoking among adults? | Smoking rates were lower among those trying to lose/maintain weight than those not trying (25% vs. 31%). The relationship between trying to lose weight and current smoking varied per age. Among respondents <30 years of age, those trying to lose weight were more likely to smoke currently, whereas older adults trying to lose weight were as likely or less likely to smoke compared with those not trying to control weight. All smokers trying to lose weight more likely desired to quit smoking. | | Wee et al., 2001 | |  | | | |
|  | 1993, 1994 NHIS | What are the risk differences in mortality, morbidity, and health behaviors of immigrant and U.S.-born groups? | Consistent with the acculturation hypothesis, immigrants’ risks of smoking and chronic condition, although substantially lower than those born in the United States, increased as U.S. residence increased. | | Singh & Siahpush, 2002 | |  | | | |
|  | 1970, 1978-80, 1987-88 Smoking Suppl | What are the patterns of smoking initiation between 1950 and 1980? | In 1950, initiation was higher for males of all ages than females; in 1965, initiation rates had declined much more for females; in 1980, no gender difference was seen. | | Lee et al., 1993 | |  | | | |
| **Blacks** | 1990, 1992, 1994, 1995, 1997-99 NHIS | What is the incidence of cancer, including lung cancer, among African Americans? | | After higher incidences and death rates than Whites for many years, the incidence rate declined by 2.7%/yr since 1992 in Black males and stabilized in females. The decline in death rates from lung cancer among Black men is a result of decreases in prevalence of smoking over the previous 30 years. | | Ghafoor et al., 2002 |  | | | |
|  | 1990-94 NHIS  92 CCS, CES | What are the differences in smoking status and number of cigarettes consumed daily between foreign- and native-born Blacks? What is the impact of demographic and socioeconomic factors of the behavior? | Native-born Blacks were more likely to be current smokers than foreign-born Blacks. Within the native-born, smoking prevalence decreased with rising education and income. Women were less likely to be smokers than men in both groups. | | King, Polednak, Bendel, & Hovey, 1999 | |  | | | |
|  | 1990-94 NHIS | What are the differences in current cigarette smoking among Black respondents? | Gender and regional factors such as social history of migration, social stress and racism, exposure to tobacco ads, variations in cultural influences, community structures, and coping strategies have an effect on cigarette smoking behavior in Blacks. | | King, Polednak, & Bendel, 1999 | |  | | | |
|  | 1990-93 NHIS | What are the effects of socioeconomic and demographic indicators on recent smoking behavior? | The highest smoking prevalence occurred in 1991. Those in the lowest income group had the highest prevalence; smoking decreased with increasing education. | | King, Grizeau, et al., 1998 | |  | | | |
| **Blacks, Whites** | 1985 NHIS | What are the independent effects of race, SES, and demographic factors on ever smoking, quitting, and heavy smoking? | The odds of ever smoking are not higher for Blacks compared with Whites when other variables are controlled. By contrast, the odds of heavy smoking for Blacks are far less than for Whites, while Blacks are significantly less likely than Whites to quit smoking regardless of SES or demographic factors. | | Novotny et al, 1988 | |  | | | |
|  | 1987, 1988 NHIS | What is the trend in the age at initiation of regular smoking by race and sex? | The overall proportion of persons who became regular smokers before ages 16, 18, 21, and 30 increased across successive birth cohorts; among Blacks, the increase occurred only before ages 21, 25, and 30. More than 80% of smokers born after 1930 began smok­ing regularly by age 21. Among the successive birth cohorts, the average age at smoking initiation decreased 2.4 years for Whites, and 1.3 years for Blacks. The average age at initiation decreased substantially for White and Black females (5.4 and 4.6 years, respectively), decreased slight­ly for White men (0.5 year), and increased slightly for Black men (0.7 year). In 1974, 38.6% of Whites and 47.1% of Blacks ages 20-24 were cur­rent smokers; by 1988, the proportions of Whites and Blacks in this age group who were current smokers had decreased to 28.5% and 24.8%, respectively. | | Giebel et al., 1991 | |  | | | |
|  | 1970, 1979-80 Smoking Suppl | How do you derive unbiased estimates of the incidence and prevalence of smoking, especially at younger ages, and their changes over time? | Reconstructing a series of estimates of “risks” actually experienced by a cohort is possible only if other prevalence fig­ures are available for the same representa­tive section of the pop­ula­tion, at different times and at different ages. | | Weinkam & Sterling, 1990 | |  |  |  |  |
|  | 1974, 1976, 1978- 80, 1983, 1985 Smoking Suppl | What are the trends in the prevalen­ce, initiation, and cessation of cigarette smoking for the U.S. population using weighted and age-standardized data? | Smoking prevalence is decreasing across all race-gender groups, although at a slower rate for women than men; differences in initiation, more than cessation, are primarily responsible for the converging of smoking prevalence rates among men and women. | | Fiore et al., 1989 | |  | | | |
|  | 1987 CC & CES | How do success rates in smoking cessation compare by sex, ethnic status, and birth cohort? | Success in quitting was independent of ethnic status and sex; population differences in smoking initiation age could produce statistical association between sex/ethnicity and smoking cessation. Population differences in smoking initiation patterns can mask similarities in cessation rates. | | McGrady & Pederson, 2002 | |  | | | |
| **Blacks, Hispanics** | 1990 HPDP Suppl  1992 CCS | What percentage of adults know the risk factors for oral cancer and recognize the signs? | | Two-thirds of respondents identified tobacco use as a risk factor for oral cancer. Lack of knowledge persisted across all groups analyzed. | | Horowitz et al., 1995 |  | | | |
| **Whites,**  **All Ages** | 1965-88 NHIS | What are the trends in cessation patterns and projected future experience in relation to age of initiation? | | The median cessation age for those who started smoking as adolescents is expected to be age 33 for males and age 37 for females; 50% of these adolescents may smoke for at least 20 years, based on a median initiation age of 16-17. Despite the decline in the median age of smokers who quit, smoking will be a long-term addiction for many adolescents who start now. | | Pierce & Gilpin, 1996 |  | | | |
| **White, Mexican American, Cuban American, Puerto Rican American** | 1987 NHIS  1982-83 Hispanic HANES | What are the trends in cigarette smoking initiation between 1982 and 1987 among Hispanics? | In general, rates of smoking initiation either declined or leveled off later for Hispanics than for Whites. The results suggest that Hispanics tended to follow the smoking trends observed among Whites. | | Escobedo et al., 1989 | |  | | | |
| **American, Canadian** | 1985 HPDP  1985 Canada Health Promotion Study | What differences in health behaviors exist between the U.S. and Canada? | Canadians smoke more than U.S. adults. | | Schoenborn & Stephens, 1988 | |  | | | |
| **Females** | 1987 CCS | What are the age patterns of cigarette smoking among females, by race? | White women initiate cigarette smoking at younger ages but are more likely to quit. | | Geronimus et al., 1993 | |  | | | |
| **Black, White**  **Females** | 2000 NHIS | What are the ethnic differences in smoking patterns among African American and White women? | Current female smokers: 18-20 age group, White 28%, Black 15%; 41-43 age group, White 28%, Black 36%. Blacks initiate smoking later than Whites in each age group. | | Moon-Howard, 2003 | |  | | | |
|  | 1985 NHIS | What are the relationships between race and health behavior for Black and White women and possible differences between rural and urban residents concerning their health behavior? | Black females are less likely to engage in primary prevent­ion behaviors yet are more likely to engage in secondary ones. The higher percentage of smok­ing among Black women is due to their lower levels of education. Urban Black females are most likely to be smokers. | | Duelberg, 1992 | |  | | | |
| **Adolescents/**  **Young Adults** | 1970, 1978-80, 1987, 1988 Smoking Suppl | Is there a relationship between knowledge of health consequences of smoking and decreases in initiation rates? | Smoking initiation in adult males declined sharply around 1950; the decline for adult females began in the mid-1960s; the rate for females ages 10-14 and 15-20 increased through the 1970s. | | Gilpin et al., 1994 | |  | | | |
| **Adolescents/ Young Adults**  **-Females** | 1970, 1978-80, 1987, 1988 Smoking Suppl | How does specific targeting of tobacco advertising to women influence smoking initiation rates in adolescent girls? | In women ages 18-20, initiation peaked in the early 1960s and steadily declined thereafter; in girls under age 18, initiation increased abruptly around 1967. | | Pierce et al., 1994 | |  | | | |
| **Adolescents**  **-Blacks** | 1992 YRBS | Can race differential in cigarette smoking prevalence be attributed to differences in selected lifestyle behaviors and demographic factors? | Selected lifestyle behaviors and demographic factors do not account for the race differential in adolescent smoking. | | Faulkner & Merritt, 1998 | |  | | | |
| **Adolescents**  **-Blacks**  **-Hispanics** | 1992 YRBS | What is the prevalence of cancer risk behaviors related to SES? | Among respondents, 63% reported two or more risk behaviors; as income level increased, respondents were less likely to smoke. | | Lowry et al., 1996 | |  | | | |
| **Adolescents/**  **Young Adults**  **-Whites**  **-Blacks**  **-Hispanics** | 1987 NHIS | Are there any patterns in age-specific initiation of cigarette smoking in relation to race/ethnicity, sex, and education attainment? | Incidence of smoking initiation increased rapidly after age 11, peaked in groups 17-19 of age, rapidly declined in groups through age 25, and gradually declined afterwards. Age-specific smoking initiation rates were generally lower among Blacks than Whites, similar between Whites and Hispanics, and appreciably higher among Black and Hispanic males than females. Compared with those graduated from high school, persons with less education were consistently more likely to start smoking during childhood and adolescence. Age and education attainment are factors consistently associated with cigarette smoking initiation among all race/ethnic groups in the United States. | | Escobedo et al., 1989 | |  | | | |
| **Young Adults**  **-Whites** | 1978-80, 1987  Smoking Suppl | At what age is regular smoking initiated? | The uptake of regular smoking occurs general­ly before age 25. However, in the 1960-62 birth cohort, less than 18% of ever smokers with at least a 12-year education did not start to smoke regularly until ages 19-24. | | Pierce et al., 1991 | |  | | | |
| **Adolescent/**  **Young Adult**  **-Blacks**  **-Other** | 1978, 1979 Smoking Suppl | What are the determinants of the decision to smoke? | Non-economic variables, such as lifetime educational attainment, marital stress, race and gender, appear to have a much larger impact than price or income on the probability and timing of initiating the smoking habit. | | Douglas & Hariharan, 1994 | |  | | | |
| **Adolescent/**  **Young Adult**  **-Hispanics**  **-Non-Hispanics**  **-Other** | 1992 YRBS | What is the prevalence of smoking among adolescents? | About half had ever smoked a whole cigarette. The percentage of those who had tried to quit declined steadily with age; use of chewing tobacco and snuff were much lower than cigarette use and highest in White males. | | Adams et al., 1995 | |  | | | |
|  | 1992 YRBS | What are the differences by ethnic group in the performance of cancer risk related lifestyle behaviors through the transition out of high school? | Hispanic-American males experienced somewhat higher risks for chewing tobacco. snuff use after the transition out of high school. | | Baranowski et al., 1999 | |  | | | |
|  | 1992 YRBS | Do health-related behaviors change at times of major life transitions and are there differences by gender? | There are significant gender differences by transition effects. Daily and heavy cigarette smoking increased during high school years. | | Cullen et al., 1999 | |  | | | |
| **Blacks, Whites** | 1970 NHIS | Are there pronounced differences in prevalence intensity, kind, and cessation of smoking related to type of employment? | There are pronounced differences in prevalence, intensity, kind, and cessation of smoking related to type of employment. Sex, race, and occupation reflect physiological, social, cultural, and economic conditions that influence the prevalence and amount of cigarette smoking. | | Sterling & Weinkam, 1976 | |  | | | |

* Specific Population can be assumed to be adult males and females, unless otherwise stated. Categories reflect the authors’ terminology used to describe their sample and does not imply consistency among population parameters.
