# Supplementary material for: Using the National Health Interview Survey to understand and address the impact of tobacco in the United States: past perspectives and future considerations
Source: Epidemiol Perspect Innov. 2008 Dec 4;5:8. doi: 10.1186/1742-5573-5-8 (PMC2627846; doi:10.1186/1742-5573-5-8)
Supplement: Additional file 7 — Analyses of NHIS Data: Cessation and Advice to Quit Smoking. [file 1742-5573-5-8-S7.doc]

# Table 7. Analyses of NHIS Data: Cessation and Advice to Quit Smoking

| **Specific Population** | **Data Source** | **Research Question** | **Reported Findings** | **Reference** |
| --- | --- | --- | --- | --- |
| **Blacks, Hispanics** | 1992 CCS | What proportion of adults receives oral cancer screening and tobacco cessation advice? | Less than 10% of adults reported oral cancer screening within past 10 years. Among smokers and smokeless tobacco users, about half had seen a dentist within the past year. One-quarter of those had been advised to quit. Heavy smokers were more likely to be advised to quit. | Martin et al., 1996 |
|  | 2005 NHIS | Do smoking-cessation treatments (advice and use of interventions) vary by race/ethnicity? | Black and Hispanic smokers continue to be less likely than White smokers to receive and use tobacco-cessation interventions. | Cokkinides et al., 2008 |
| **Adults** | 1965-1993 NHIS | What is the relationship between prevalence and cessation rates? | Cessation rates have been reasonably stable over the last 15 years. Prevalence issues are more likely to be due to initiation rates vs. cessation rates. Although prevalence of smoking among adults will continue to decline even with initiation rates rising to 35%, the eventual decline will be modest due to 27% initiation rate by youth. | Mendez et al., 1998 |
|  | 1976-1980, 1983,  1985, 1987-1993 NHIS | What is the potential impact of cigarette price increases on minority and lower income populations? | Lower income, minority, and younger populations would be more likely to reduce or quit smoking in response to a price increase in cigarettes. | CDC, 1998; 29; 605 |
|  | 2001 NHIS | What is the estimated effect of provider advice in routine clinical contacts on patient smoking cessation outcome? | Provider advice doubles the chances of success in self-reported smoking cessation by their patients. The probability of quitting by the end of the 12-month reference period increased from 6.9% to 14.7%, an effect that is of both statistical (*p*<.001) and clinical significance. | Bao et al, 2006 |
|  | 1997-2002 NHIS | What is the result of using simulation models to investigate the costs and benefits of covering smoking cessation intervention from insurers’ and employers’ perspectives? | By the final simulation year, insurers had benefit-cost ratios of 0.56 to 1.67 with per member per month costs of -$0.22 to $0.43. The earliest year at which savings were achieved for insurers was year 8. Employers saw benefit-cost ratios of 1.88 to 5.58 by the final simulation year with per member per month costs of -$1.23 to -$0.15. Employers achieved savings as early as year 3 and as late as year 8. Models were sensitive to the rate at which population members were assumed to exit the insurer or employer. | Levy, 2006 |
|  | 1991 HPDP  1990 NHEFS NHANES I Epidemiologic Follow-up | What is the potential effect of making nicotine gum available over-the-counter? | Three million or more people per year would use nicotine gum. | Oster et al., 1996 |
| **Adolescents and Adults** | 1965, 66, 70, 78-80, 83, 85, 87-88, 90-91 Smoking Supplement | Is news media coverage of smoking and health issues associated with changes in smoking behavior in the United States? | From 1950 to the early 1980s annual incidence of cessation mirrored the pattern of news media coverage of smoking and health issues, particularly for middle aged smokers. Print media coverage correlated with adult quitting behavior but not adolescent initiation behavior. | Pierce & Gilpin, 2001 |
| **Adults, Ages 25 or Older** | 2001 NHIS | Is there an association between selected sociodemographic and tobacco-related factors and smoking cessation assistance? | Smokers were less likely to report assistance if they were younger, Black, or had a high or middle level of socioeconomic disadvantage. Being married, attempting to quit in the past 12 months, consuming more tobacco, increased age, and greater socioeconomic advantage (higher education and income and health insurance) were associated with receiving assistance to quit smoking. | Browning et al., 2008 |
| **Older Adults** | 1990 HPDP | What are the national point prevalence profiles and what is the association of age, health status, and health beliefs of older adults who ever smoked or currently smoke? | Among adults above age 54, 53% smoked in the past, and 17% smoked in 1990. Among smokers, 61% tried to quit and 36% noted that their physicians never advised them to quit. | Ruchlin, 1999 |
| **Blacks, Whites** | 1987 CC & CES | How do success rates in smoking cessation compare by sex, ethnic status, and birth cohort? | Success in quitting was independent of ethnic status, sex, and smoking initiation age. Population differences in smoking initiation patterns can mask similarities in cessation rates. | McGrady & Pederson, 2002 |
|  | 1965-66, 1970, 1978-80, 1983, 1985, 1987-88, 1990-92 Smoking Supplements | Can changes in the incidence of successful quitting among population subgroups, a new measure of cessation, inform policy makers? | Overall, incidence increased over fivefold, from <1% in 1950 to a low of 5% in 1990.Younger smokers appeared to increase quitting markedly in the 1970s. Quitting patterns in middle-aged Blacks were similar to patterns in Whites, although at a much reduced rate. Younger Blacks had low quitting incidence until 1989. Incidence differed by education attainment; regardless of age, during the 1970s and 1980s; those with some college increased their quitting incidence markedly. | Gilpin & Pierce, 2002 |
|  | 1985 NHIS | What are the independent effects of race, socioeconomic status (SES), and demographic factors on ever smoking, quitting, and heavy smoking? | The odds of ever smoking are not higher for Blacks compared with Whites when other variables are controlled. The odds of heavy smoking for Blacks are far less than for Whites, while Blacks are significantly less likely than Whites to quit smoking, regardless of SES or demographic factors. | Novotny et al., 1988 |
| **Black**  **Females** | 1987 CCS | Do age patterns of smoking vary by race? | White women initiate smoking at younger ages but are more likely to quit. | Geronimus et al., 1993 |
| **Blacks, Hispanics** | 1965-1994 NHIS | What are the patterns of cigarette smoking and smoking cessation among older adults in the United States? | Smoking prevalence for those age 65+ declined from 1965 to 1994 and was lower among older than younger adults. Prevalence of cessation rose with educational level and was higher for men than women and for Whites than Blacks. No racial differences were found among women. Older White and Hispanic men were significantly more likely to be former smokers than older Black men. | Husten et al., 1997 |
| **Blacks, Hispanics, Asians, Native Americans** | 1991 HPDP | What conclusions can be drawn from a survey on counseling? | Among smokers, 37.2% reported having received advice to quit in the past year from a health care professional. | CDC, OSH, 1993; 44; 854 |
| **Whites** | 1965-88 NHIS | What are the trends in cessation patterns and how are can these project future experience? | The median cessation age for those who started smoking as adolescents is expected to be age 33 for men and age 37 for women; 50% of these adolescents may smoke for at least 20 years, based on a median initiation age of 16-17. Despite the decline in the median age of smokers who quit, smoking will be a long-term addiction for many adolescents who start now. | Pierce & Gilpin, 1996 |
| **Non-Whites** | 1992 CCS | How do smokers in three age groups differ in background characteristics, smoking behavior, or attitudes? | Oldest smokers of age >50 consistently had attitudes least favorable to smoking cessation; smokers ages 30-49 were more likely to be in contemplation stage. | Clark et al., 1997 |
| **Hispanics, non-Hispanics** | 1990-91  HPDP | How do those who smoke cigarettes every day or some days compare with those who now smoke cigarettes not at all or some days? | Among respondents, 5.7% quit smoking and maintained abstinence for at least 1 month. Among those who were daily smokers, college graduates, and persons at/above the poverty level were more likely than those with less education and below the poverty level to abstain from smoking 1 month or more. | CDC, OSH, 1993; 26; 504 |
| **Californians** | 1998 Draft 2010 Objectives | What changes in smoking initiation and cessation are needed to realize the 2010 objective of 13% national adult smoking prevalence in California? | Except by plausible decreases in initiation and increases in cessation, the draft objective is virtually unattainable. | Mendez & Warner, 2000 |
| **Family Medicine Residents** | 1985 NHIS  1985 Family Medicine  Resident Survey | How do health-promoting behaviors of family medicine residents compare with those of the general population? | Less than 5% of the family medicine residents were currently smoking vs. 28% in NHIS; 86.4% of female resi­dents and 77.4% of male residents had never smoked compared with their respective counterparts′ rates of 55.4% and 45.3%. Residents were more likely to counsel patients about such health risks if they were nonsmokers. Residents were modeling health-promoting behaviors at a significantly higher rate than their national counterparts. | Young, 1988 |
| **Adult Males** | 1998 NHIS | What is the association between snuff use and smoking in a representative sample of U.S. men? | Some may use snuff to quit smoking, but more commonly men switch from snuff use to smoking. Some smokers may use snuff to supplement their nicotine intake, smokers who also use snuff are more likely than non-users to try to quit smoking but tend to have less success. | Tomar, 2002 |
|  | 2000 NHIS | What is the estimated use and effectiveness of smokeless tobacco as a smoking cessation aid among U.S. men? | An estimated 359,000 men switched to smokeless tobacco in their most recent quit attempt. This method had the highest proportion of successes among those attempting it (73%). | Rodu & Phillips, 2008 |

* Study Population can be assumed to be adult males and females, unless otherwise stated. Categories reflect the authors’ terminology used to describe their sample and does not imply consistency among population parameters.
