# Supplementary material for: Using the National Health Interview Survey to understand and address the impact of tobacco in the United States: past perspectives and future considerations
Source: Epidemiol Perspect Innov. 2008 Dec 4;5:8. doi: 10.1186/1742-5573-5-8 (PMC2627846; doi:10.1186/1742-5573-5-8)
Supplement: Additional file 8 — Analyses of NHIS Data: Health Care Practices Regarding Smoking. [file 1742-5573-5-8-S8.doc]

# Table 8. Analyses of NHIS Data: Health Care Practices Regarding Smoking

| **Specific Population** | **Data Source** | **Research Question** | **Reported Findings** | **Reference** |
| --- | --- | --- | --- | --- |
| **Adults** | 1992 CCS | How can examinations for oral cancer among U.S. adults be characterized? | Percentages of tobacco smokers who ever had an oral cancer examination: current smokers, 13.0%; former smokers, 16.7%, never smokers, 13.9%.  Percentages of smokeless tobacco users who ever had an oral cancer examination: current users 11.2%, former users 13.8%, and never users 14.5%. | CDC, 1994; 11; 198 |
|  | 1977 H-1 (Healthy Habits) Supplement | What is the possible association between health practices and health care utilization in a national sample? | The never smokers reported fewer visits to dentist. The trends for smoking cigarette support the association of lower utilization with better health practices. | Wetzler & Cruess, 1985 |
| **Blacks, Hispanics** | 1987 CRFS  1987 NMES | What medical costs can be attributed to smoking? | Smoking affects medical expenditures to a significant and substantial degree, translated currently to $50 billion per year in medical care expenditures in the United States. | Miller et al., 1999 |
|  | 1992 CCS | What factors are associated with having an oral cancer examination among U.S. adults age 40 and older? | Among the respondents, 17.1% with knowledge about risk factors of tobacco use have had an oral cancer screening examination. | Horowitz & Nourjah, 1996 |
| **Whites** | 1970 NHIS | What is the difference in remaining lifetime hospital days (RLHD) between ever smokers and never smokers? | There is no consistent increase in RLHD for ever smokers. Male ever smokers older than age 44 and female smokers older than age 38 can expect fewer RLHDs than never smokers. | Weinkam et al., 1987 |
| **Females** | 1990 HPDP  1990 Ontario Health Survey | Is healthy lifestyle associated with use of preventive services? | Compared with Canadians, higher educated groups in the United States were 40% more likely to have a healthy lifestyle, such as not having smoked. | Hofer & Katz, 1996 |
|  | 1990-94 NHIS | What is the association of smoking status with breast and cervical cancer screening? | Women who smoked ≥1 pack per day were less likely to have had a mammogram or Pap test. Former smokers often had significantly higher screening rates than never smokers. | Rakowski et al., 1999 |
| **Adolescents/**  **Young Adults** | 1992 YRBS | What is the association between smoking and other high-risk behaviors among adolescents? | Though no causal links were found between smoking and other high-risk behaviors, data show a consistent association between smoking and such behavior among adolescents. | Willard & Schoenborn, 1995 |
| **Households with Knowledge of Radon** | 1993-94 Year 2000 Supplement | What is the likelihood of radon testing when a residential smoker is in the dwelling? | Households with a residential smoker were significantly less likely to have been tested for radon than those without a smoker (5.9% vs. 7.1%). | CDC, 1999; 31; 683 |

* Specific Population can be assumed to be adult males and females, unless otherwise stated. Categories reflect the authors’ terminology used to describe their sample and does not imply consistency among population parameters.
