# Supplementary material for: Using the National Health Interview Survey to understand and address the impact of tobacco in the United States: past perspectives and future considerations
Source: Epidemiol Perspect Innov. 2008 Dec 4;5:8. doi: 10.1186/1742-5573-5-8 (PMC2627846; doi:10.1186/1742-5573-5-8)
Supplement: Additional file 9 — Analyses of NHIS Data: Health Consequences. [file 1742-5573-5-8-S9.doc]

# Table 9. Analyses of NHIS Data: Health Consequences

| **Specific Population** | **Data Source** | **Research Question** | **Reported Findings** | **Reference** |
| --- | --- | --- | --- | --- |
| **Cigarettes** | | | | |
| **Adults** | 1970 NHIS Smoking Supplement | What is the evidence regarding confounding between smoking and occupation and what are the consequences of not controlling for this confounding? | Well-planned further studies are needed to disentangle the effects of occupation and smoking from each other. | Sterling & Weinkam, 1990 |
|  | 1987 CES  1988 OHS  1990 HPDP  86-91 linked NHIS/ Nat’l Death Index (NDI) 1987 NHIS Cancer Control | Can factors other than race help explain differences in cancer mortality? | Caucasian-American mortality was higher, in part because of a high prevalence and quantity of smoking. | Rogers et al., 1996 |
| **Adolescents/ Young Adults** | 1992 YRBS | What is the relationship between unhealthy behaviors and smoking in adolescents and young adults? | Though no causal links were found between smoking and other high-risk behaviors, data show a consistent association between smoking and such behaviors among adolescents. | Willard & Schoenborn, 1995 |
| **Blacks, Whites, Other**  **Ages <1–Adult** | 1974-87 NHIS | What is the smoking-attributable mortality (SAM) and years of potential life lost (YPLL) in the U.S. in 1988? | Smoking is a leading cause of diseases associated with premature mortality in the U.S. In 1988, 434,000 deaths and 1,199,000 YPLL before age 65 were attributable to cigarette smoking. SAM and YPLL rates varied by race and sex. | Schultz, 1991 |
| **Hispanics** | 1979, 1980 NHIS | What is the evidence on smoking and lung cancer among Hispanics? | The study found a notable sex difference in smoking rates among Hispanics, with males reporting smoking as frequently or more than White men, and Hispanic women reporting rates considerably lower than those of White women. This pattern held true across Hispanic subgroups, including country of origin. | Marcus & Crane, 1985 |
| **Hispanics, Non-Hispanic Whites** | 1991 NHIS | How is acculturation related to smoking, drinking alcohol, leisure time exercise activity, and BMI? | Hispanics’ current smoker ratio is 20.9% (16.2% F, 26.0% M) vs. 26.5% of non-Hispanic Whites (25.1% F, 28.0% M). | Abraido-Lanza et al., 2005 |
| **Hispanics, Blacks, Other** | 1987 NMES  1987 CRFS | What are the estimated medical costs attributable to cigarette smoking using an economic model? | Cigarette smoking affects medical expenditures to a significant and substantial degree: more than $50 billion per year in medical care expenditures in the United States, most paid through the public sector. | Miller et al., 1999 |
| **Blacks, Whites**  **Age 45+** | 1983-87 NHIS | What is the prevalence of heart and lung diseases and risk factors analyzed by U.S region and level of urbanization? | Geographic variations in self-reported disease prevalence by region and urbanization were not consistent across age or sex groups and did not consistently parallel patterns reported for mortality, with the exception of high rates in the nonmetropolitan South and relatively low rates in some groups in SMSAs outside central cities. | Gillum, 1994 |
| **Blacks, Whites**  **Male** | 1970, 79-80 NHIS  Smoking Supplements | How do lung cancer risk factors of smoking prevalence, amount smoked, and age of smoking initiation compare between Blacks and Whites, as well as occupational category? | Black smokers smoked significantly less than Whites and tended to start smoking later in life. Increased lung cancer incidence among Blacks relative to Whites may be due more to occupational differences than to differences in Black and White smoking. | Sterling & Weinkam, 1989 |
| **Whites** | 1985 HPDP 1986 NMFS 1987 CES | What are the life expectancies of cigarette smokers and nonsmokers in the United States? | In general, life expectancies were higher for never smokers than for former smokers, and higher for former smokers than for current smokers. Heavy smokers had lower life expectancies than those in all other smoking statuses. | Rogers & Powell-Griner, 1991 |
| **Smokeless Tobacco** | | | | |
| **Adults** | 1987 CES  1986 Nat’l Mortality Followback Survey | Does smokeless tobacco increase the risk of oral cancer or cancer of digestive organs? | This study calculated the relative risk of all cancer, oral cancer, and cancer of the digestive organs for use of smokeless tobacco and adjusted for smoking, drinking, occupation, sex, race and age. | Sterling et al., 1992 |
| **Environmental Tobacco Smoke** | | | | |
| **Adults** | 1986 NMFS  1987 CES  1987, 1992 NHIS  1992 CCS | How should the number of lung cancer deaths due to environmental tobacco smoke (ETS) exposure among the 1992 U.S. never-smoking population be estimated? | The linear extrapolation model estimated that five male and six female excess lung cancer deaths due to ETS exposure would be expected in the 1992 U.S. population of more than 52 million never smokers ages 35 and older. | Rosenbaum, Sterling, & Weinkam, 1998 |

* Specific Population can be assumed to be adult males and females, unless otherwise stated. Categories reflect the authors’ terminology used to describe their sample and does not imply consistency among population parameters.
