# Supplementary material for: Using the National Health Interview Survey to understand and address the impact of tobacco in the United States: past perspectives and future considerations
Source: Epidemiol Perspect Innov. 2008 Dec 4;5:8. doi: 10.1186/1742-5573-5-8 (PMC2627846; doi:10.1186/1742-5573-5-8)
Supplement: Additional file 10 — Analyses of NHIS Data: Secondhand Smoke. [file 1742-5573-5-8-S10.doc]

# Table 10. Analyses of NHIS Data: Secondhand Smoke

| **Specific Population** | **Data Source** | **Research Question** | **Reported Findings** | **Reference** |
| --- | --- | --- | --- | --- |
| **Adults** | 1986 NMFS  1987 CES  1987 NHIS  1992 CCS | What is the estimated number of lung cancer deaths due to environmental tobacco smoke (ETS) exposure among the 1992 U.S. never-smoking population? | In the 1992 U.S. population of >52 million never-smokers ages >35, it is estimated that five male and six female excess lung cancer deaths per 100,000 are due to ETS exposure. | Rosenbaum, Sterling & Weinkam, 1998 |
|  | 1987 CES | What is the effect of common courtesy on smoking behavior in public places? | Among smokers, 47% light up in a public place without asking others if they mind; only 4% of nonsmokers ask others not to smoke. | Davis, Boyd, & Schoenborn, 1990 |
|  | 1991 NHIS | What is the effect of ETS exposure in the home and worksite on the health of U.S. adults? | Among never-smokers, people exposed to ETS were more likely to report 1+ days each of restricted activity, bed confinement, and work absence than people without such exposure. | Mannino, Siegel, et al., 1997 |
|  | 1970, 1991 NHIS | Do toxic occupational exposures confound data on ETS? | Exposure to toxic chemicals confounds the ETS data. | Sterling, Glicksman, et al.,1996 |
|  | 1988 OHS | How many workers experience discomfort in the work place due to ETS? | Among nonsmokers, 43.5% reported some or moderate discomfort, and 15.7% reported great discomfort. | CDC. 1992; 20; 351 |
| **Non-White Children and Adults** | 1976-80 NHIS | What is the effect in terms of respiratory restricted days for smokers and for children and spouses of heavy smokers? | Smokers have a 55-75% excess in respiratory restricted days; living with heavy smokers increased respiratory restricted days of spouses by 20% and bed days of children by 20%. | Ostro, 1989 |
| **Children Under Age 18** | 1994 Year 2000 Objectives | What is the prevalence and pattern of ETS exposure in U.S. homes with children? | Smoking occurs every day in smokers’ homes. Visitors are an additional source of ETS in homes, including nonsmokers’ homes. | Schuster, Franke, & Pham, 2002 |
| **Children Under Age 16** | 1970 NHIS | What impact does living in a home with cigarette smokers have on children’s health? | Children in families with no smokers had an average of 1.1 fewer restricted-activity days and 0.8 fewer bed-disability days per year than did children in families with two smokers. Children in families that smoked >45 cigarettes a day had 1.9 more restricted-activity days and 0.9 more bed-disability days due to acute respiratory conditions than did children in no-smoker families. | Bonham & Wilson, 1981 |
| **Children Ages 7-17** | 1988 CH | What health and social factors are associated with early school retention of U.S. children? | Factor of household smoking independently associated with increased risk of repeating kindergarten and first grade was OR 1.4, CI 1.1, 1.7. | Byrd & Weitzman, 1994 |
| **Children Under Age 10** | 1991 NHIS | What is the effect of ETS exposure on children’s health? | Exposed children had 21% more days of restricted activity, 31% more days of bed confinement, and 39% more days of school absence. | Mannino, Siegal, et al., 1996 |
| **Children, Ages 5-17, With disability** | 1994, 1995 NHIS-D  1994 Year 2000 Objectives | What is the effect of ETS exposure on children with disabilities? | Children exposed to ETS at home: 48% with any limitation in self-care and 49% with a mobility limitation compared with 33% without limitation in function. Exposure to ETS increased with the severity of learning disability. | Hogan, Rogers, and Msall, 2000 |
| **Children Ages 0-4** | 1999, 2001 Medical Expenditure Panel Survey (MEPS)  1999, 2001 NHIS | What is the estimated annual excess health service use, healthcare expenditures, and disability bed days for respiratory conditions associated with young children’s exposure to smoking in the home in the United States? | Indoor smoking is associated in this age group with (1) an estimated $117 in additional healthcare expenditures for respiratory conditions for each exposed child; (2) an 8% increase in the probability of having a bed day because of respiratory illness; (3) a 5% increase in the probability of ED visits; (4) and a 3% increase in the probability of inpatient use. | Hill & Liang, 2008 |

* Specific Population can be assumed to be adult males and females, unless otherwise stated. Categories reflect the authors’ terminology used to describe their sample and does not imply consistency among population parameters.
