# Supplementary material for: Using the National Health Interview Survey to understand and address the impact of tobacco in the United States: past perspectives and future considerations
Source: Epidemiol Perspect Innov. 2008 Dec 4;5:8. doi: 10.1186/1742-5573-5-8 (PMC2627846; doi:10.1186/1742-5573-5-8)
Supplement: Additional file 11 — Analyses of NHIS Data: Smokeless Tobacco. [file 1742-5573-5-8-S11.doc]

Table 11. Analyses of NHIS Data: Smokeless Tobacco

| **Specific Population** | **Data Source** | **Research Question** | **Reported Findings** | **Reference** |
| --- | --- | --- | --- | --- |
| **Adults** | 1965-66, 1970, 1974, 1978-80, 1983, 1987-88, 1990-91, NHIS 1984-92 BRFSS | How can surveillance data on chewing tobacco and snuff be used to guide research initiatives, intervention programs, and policy decisions? | The prevalence of chewing tobacco use and snuff use increased substantially between 1970 and 1987 among White men ≤ 34 years of age; this trend was not observed among Black men or women or White women. The prevalence of use was found to vary by age and ethnicity over the periods studied. | Giovino et al., 1994 |
|  | 1987 CEC | What is the prevalence of smoking and other tobacco use? | Overall, 4% of males chewed tobacco and 3.1% used snuff; 6.1% used both. Of men ages 18-24, 8.9% used either chewing tobacco or snuff or both, compared with 5.3% of men ages 25-64; smokeless tobacco use was also higher in men ≥75 years (7.9%).Use of smokeless tobacco was rare among women: 0.3% used chewing tobacco and 0.5% used snuff. | Boyd, 1989 |
|  | 1991 HPDP | What are the trends in the prevalence of use of smokeless tobacco products in a representative sample of the U.S. population? | In 1991, an estimated 5.3 million U.S. adults were current users of smokeless tobacco, including 4.8 million men and 533,000 women. The prevalence of smokeless tobacco use was substantially higher among men for all categories of comparison. Prevalence of use varied by age, ethnicity, education, region, urban/rural, and poverty status. | CDC, 1993; 14; 263 |
|  | 1987 CES  1986 NMFS | Does smokeless tobacco increase the risk of oral cancer or cancer of digestive organs? | Use of smokeless tobacco is not associated with increased risk of oral cancer or cancer of the digestive organs. | Sterling et al., 2002 |
| **Adolescents and Young Adults** | 1992 YRBS | What is the prevalence of selected self-reported health-risk behaviors among persons ages 12-21 years? | Current use of smokeless tobacco was significantly higher among the older age groups (ages 14-17 and 18-21). | CDC, 1994; 13; 231 |
|  | 1992 YRBS | What is the prevalence of selected high-risk behaviors in the U.S. adolescent population? | The prevalence of use of chewing tobacco and snuff was much lower than for cigarette smoking. | Adams et al., 1995 |
|  | 1992 YRBS | What differences by ethnic group exist in the performance of cancer risk-related lifestyle behaviors through the transition out of high school? | For males, there was a slight increase in chewing tobacco use during the high school years, a step increase at the transition, and a decline after the transition. Hispanic Americans had a significantly increased likelihood of using chewing tobacco at the intercept. African Americans showed the highest use increase during high school. (Females were excluded from this analysis.) | Baranowski et al., 1999 |
|  | 1992 YRBS | What chronic disease risk behaviors exist among male and female youth ages 14-21 during the transition out of high school? | Snuff use decreased during high school for females but was maintained through the transition years for males. | Cullen et al., 1999 |
| **Males, Ages 18-34** | 1987 CCS | What are the noncausal and causal patterns of smokeless tobacco and cigarette use and the prevalence of nongateway and possible gateway pattern of smokeless tobacco use? | Of those ages 23-34 who had ever used smokeless tobacco with or without cigarettes, 77.2% were classifiable as non-gateway users, in that 35.0% had only used smokeless tobacco and 42.2% had used cigarettes first. Cigarette use in younger cohorts was less common, despite increased smokeless tobacco use. Those who smoked before smokeless tobacco use were 2.1 times more likely to have quit smoking than cigarette-only users. | Kozlowski et al., 2003 |
| **Blacks, Hispanics** | 1992 CCS | What is the proportion of Black and Hispanic U.S. adults who received oral cancer screening and tobacco cessation advice? | Less than 10% of adults reported oral cancer screening within the past 10 years. Among smokers and smokeless tobacco users, about half had seen a dentist within the past year, one-quarter of those had been advised to quit. Heavy smokers were more likely to be advised to quit. | Martin et al., 1996 |
| **Blacks, Hispanics**  **Age 40+** | 1992 CCS | What factors are associated with having an oral cancer examination among U.S. adults 40 years of age or older? | Respondents who were above the poverty level, White, non-Hispanic, ages 40-64, and who had more than a high school education and a higher level of knowledge about risk factors for oral cancer were more likely to have had an oral cancer examination. | Horowitz & Nourjah, 1996 |
| **Blacks, Hispanic Males** | 1987 NHIS | Who is at highest risk of using snuff based on their characteristics? | The model is used to estimate proportions of men 18 years of age and older who use snuff for each of the categories of a group of descriptive variables. The strengths and limitations of these estimates are discussed. | Graubard & Korn, 1999 |
|  | 1998 NHIS | What is the association between snuff use and smoking in U.S. men? | Some may use snuff to quit smoking, but more commonly switch from snuff use to smoking. Some smokers may use snuff to supplement their nicotine intake; smokers who also use snuff are more likely than non-users to try to quit smoking but tend to have less success. | Tomar, 2002 |

* Specific Population can be assumed to be adult males and females, unless otherwise stated. Categories reflect the authors’ terminology used to describe their sample and does not imply consistency among population parameters.
